# Waste-Derived Catalysts for Water Electrolysis: Circular Economy-Driven Sustainable Green Hydrogen Energy

**DOI:** 10.1007/s40820-022-00974-7

**Published:** 2022-12-01

**Authors:** Zhijie Chen, Sining Yun, Lan Wu, Jiaqi Zhang, Xingdong Shi, Wei Wei, Yiwen Liu, Renji Zheng, Ning Han, Bing-Jie Ni

**Affiliations:** 1 Centre for Technology in Water and Wastewater (CTWW), School of Civil and Environmental Engineering, University of Technology Sydney, Ultimo, NSW 2007 Australia; 2 Functional Materials Laboratory (FML), School of Materials Science and Engineering, Xi’an University of Architecture and Technology, Xi’an, 710055 People’s Republic of China; 3 School of Minerals Processing and Bioengineering, Central South University, Changsha, 410083 People’s Republic of China; 4 Department of Materials Engineering, KU Leuven, 3001 Louvain, Belgium

**Keywords:** Waste-derived electrocatalysts, Water splitting, Sustainable hydrogen energy, Catalyst design, Circular economy

## Abstract

The sustainable production of green hydrogen via water electrolysis necessitates cost-effective electrocatalysts. By following the circular economy principle, the utilization of waste-derived catalysts significantly promotes the sustainable development of green hydrogen energy. Currently, diverse waste-derived catalysts have exhibited excellent catalytic performance toward hydrogen evolution reaction (HER), oxygen evolution reaction (OER), and overall water electrolysis (OWE). Herein, we systematically examine recent achievements in waste-derived electrocatalysts for water electrolysis. The general principles of water electrolysis and design principles of efficient electrocatalysts are discussed, followed by the illustration of current strategies for transforming wastes into electrocatalysts. Then, applications of waste-derived catalysts (i.e., carbon-based catalysts, transitional metal-based catalysts, and carbon-based heterostructure catalysts) in HER, OER, and OWE are reviewed successively. An emphasis is put on correlating the catalysts’ structure–performance relationship. Also, challenges and research directions in this booming field are finally highlighted. This review would provide useful insights into the design, synthesis, and applications of waste-derived electrocatalysts, and thus accelerate the development of the circular economy-driven green hydrogen energy scheme.

## Highlights

- Critical strategies for converting wastes to catalysts are summarized.
- Applications of waste-derived catalysts in hydrogen evolution reaction, oxygen evolution reaction, and overall water electrolysis are comprehensively reviewed.
- Perspectives in the development of waste-derived catalysts are analyzed.

## Graphical abstract

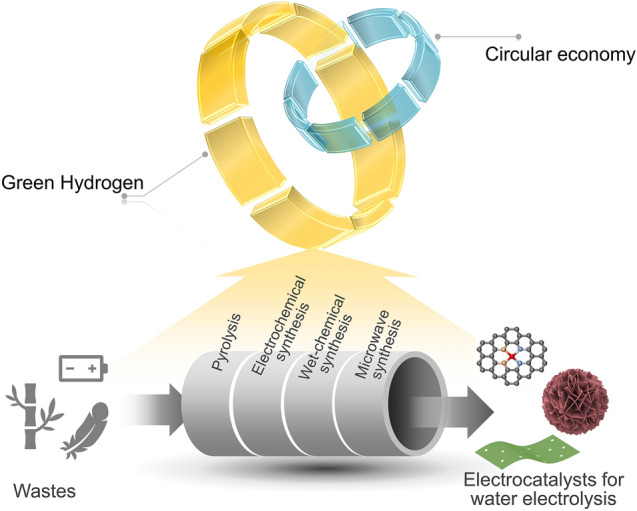

## Introduction

The utilization of traditional carbon-based fuels (e.g., natural gas, coal, oil) has given rise to serious concerns about environmental pollution and climate change [1, 2]. Additionally, the ever-climbing global energy demand is essential to sustain the development of our human society. As such, it is imperative to explore sustainable and clean energy systems to meet these energy-related challenges. Featuring zero carbon footprint, earth abundance, and high gravimetric energy density, hydrogen fuel is one of the most promising candidates to revolutionize the global energy system [3–5]. The complete industrial chain of hydrogen energy contains hydrogen production, storage, transportation, and application. A prerequisite for the sustainable development of hydrogen economy is the large-scale and clean production of hydrogen gas. Currently, conventional fossil fuels are responsible for the majority of H_2_ production, and about 71.27% of H_2_ is generated from natural gas, 27.27% from coal, 0.7% from petroleum, and the remaining 0.7% from water splitting. However, fossil reformation-based hydrogen production techniques are neither renewable nor carbon neutral as the production process involves high greenhouse gas footprints [6]. Hence, water electrolysis, which only involves the conversion of hydrogen and oxygen elements has attracted broad interest in the world [7, 8]. Although water electrolysis attains a high technology readiness level (9–10), the relatively low energy efficiency (61–82%), and high levelized cost of hydrogen ($4.78 − 5.84/kg H_2_, alkaline water electrolyzers) remain great challenges for the large-scale industrial application of water electrolysis technique [9].

Theoretically, a low thermodynamic potential of 1.23 V (at standard conditions) is needed to drive the water-splitting process [10]. Nevertheless, a considerable overpotential (*η*) is generally required for practical water electrolysis due to the system hindrance and sluggish reaction kinetics [11]. To reduce energy consumption, efforts have been made to advance high-performance electrocatalysts. Although precious metal-based catalysts (e.g., IrO_2_, RuO_2_, Pt, and Pd) exhibit high catalytic activities and durability for oxygen evolution reaction (OER) and hydrogen evolution reaction (HER), their high cost profoundly restrains their industrial applications [12, 13]. Surprisingly, many well-designed earth-abundant transitional metals (e.g., Ni, Fe, Mn, Cu, Co, Mo) and carbon-based materials also show high performance for OER, HER, and overall water electrolysis (OWE) [14–18]. Electrocatalysts with diverse structural features have gained great interest, such as metal–organic frameworks [19, 20], covalent-organic frameworks [21], two-dimensional (2D) materials [22], and hierarchically structured materials [23, 24]. The implementation of these low-cost electrocatalysts would largely cut the running cost of water electrolysis systems.

Of note, the aforementioned electroactive transitional metals and carbon are rich in typical wastes, such as electronic wastes, biowastes, and wastewater. From a circular economy perspective, reutilizing these wastes in the development of new products can achieve the close-loop utilization of substances, which would not only reduce the cost of preparing new products but also benefit the waste management system [25–28]. Compared with linear and recycling economy approaches, the circular economy route could reduce resource market dependence and lowers waste disposal costs. Additionally, it is suggested that the implementation of a circular economy in all sectors can help to limit carbon emissions by 45% by 2030, and to achieve carbon neutrality by 2050 [29]. Thus, developing functional materials from wastes is a sensible way to realize the circular economy and minimize the carbon footprint of materials preparation [30–32]. Recently, synthesizing electrocatalysts from wastes has gained increasing scientific attention thanks to the huge economic and environmental benefits [33–37]. For example, Cao and coworkers employed bean sprouts to design a N, S self-doped porous carbon catalyst for HER via pyrolysis [38]. The obtained carbon catalyst exhibits an acceptable HER activity (*η*_10_ = 413 mV, Tafel slope = 98 mV dec^−1^) in acidic media. Aside from the pyrolysis method which is usually used for converting biowastes into carbon-based catalysts, other sophisticated methods like electrochemical synthesis [39], wet-chemical synthesis [40], and microwave synthesis [41] are also capable of constructing waste-derived electrocatalysts for water electrolysis. Generally, there are three categories of electrocatalysts derived from various wastes, namely carbon-based materials (mainly refer to pure carbon and heteroatom-doped carbon materials), transitional metal-based catalysts, and carbon-based composite catalysts.

**Scheme 1:**
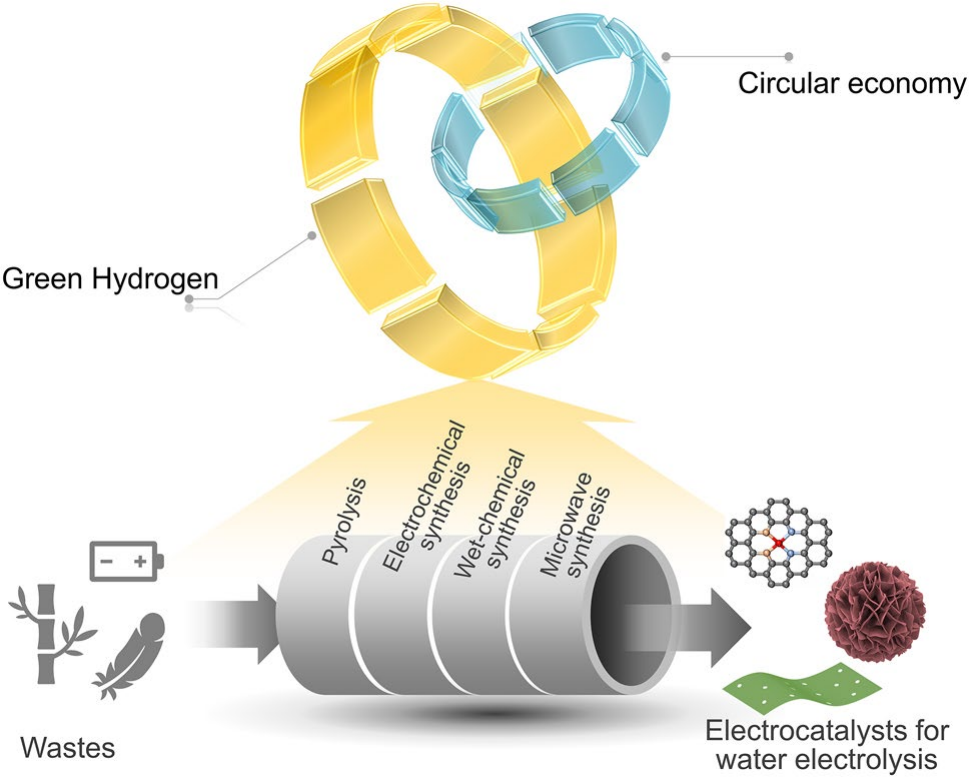
Diagram of circular economy-driven green hydrogen energy assisted by waste-derived electrocatalysts for water electrolysis

To ameliorate the catalytic performance of waste-derived catalysts, diverse strategies have been performed to regulate the physicochemical and electronic properties of catalysts, such as heteroatom doping, nanostructure control, defect/vacancy engineering, and heterostructure construction [18, 42–44]. Many engineered waste-derived catalysts exhibit good performance for HER, OER, and OWE, and some of them even outperform noble metal-based counterparts [45–49]. Hence, waste-derived efficient electrocatalysts for water electrolysis can promote the circular economy-driven green hydrogen energy system (Scheme 1). Currently, a comprehensive review of the speedily flourishing applications of waste-derived electrocatalysts in water electrolysis is still lacking. Accordingly, it is emergency to systematically summarize remarkable breakthroughs in waste-derived water electrolysis catalysts for guiding future research.

Herein, we comprehensively summarize recent achievements in applying waste-derived electrocatalysts for water electrolysis. The general principles of water electrolysis and high-performance electrocatalyst design are analyzed. Then, we introduce the main strategies for transforming wastes into catalysts, such as pyrolysis, electrochemical synthesis, wet-chemical synthesis, as well as microwave synthesis and beyond. Consequently, the applications of waste-derived carbon-based catalysts, transitional metal-based catalysts, and carbon-based heterostructural catalysts in HER, OER, and OWE are detailed separately. The catalysts’ structure–catalytic performance relationship is emphasized. At last, perspectives in this field are also pointed out. We hope this timely review would provide guidance to the design of waste-derived high-performance electrocatalysts for water electrolysis, and stimulate further studies on the development of low-cost green hydrogen production.

## General Principles of Water Electrolysis and Electrocatalyst Design

**Fig. 1:**
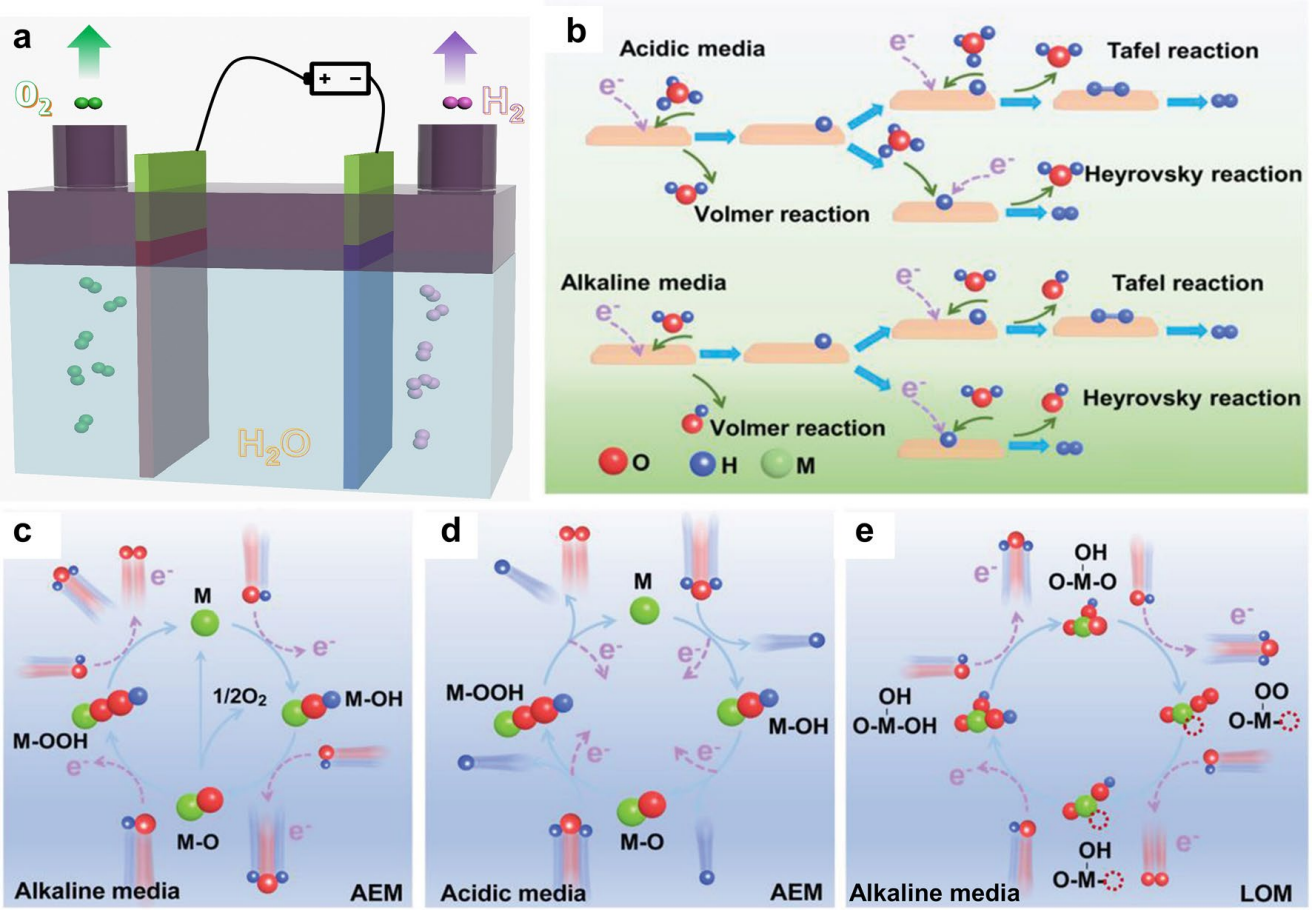
**a** Illustration of the water electrolyzer. **b** HER mechanisms in acidic and alkaline electrolytes. **c** Adsorbate evolution mechanism (AEM) for alkaline OER. **d** AEM for acidic OER. **e** Lattice oxygen participation mechanism (LOM) for alkaline OER, the dotted red circle represents the oxygen vacancy [50]. Copyright 2022, Wiley–VCH

Water electrolysis involves the splitting of H_2_O molecules into H_2_ and O_2_ gases under potential biases (Fig. 1a). The hydrogen gas production efficiency is influenced by the electrolyzer systems, including electrolytes, catalysts/electrodes, applied potentials, etc. Herein, the general principles of water electrolysis and design principles of efficient catalysts are discussed to provide an overview of the water electrolysis system.

### General Principles of Water Electrolysis

Water electrolysis consists of HER at the cathode and OER at the anode. Both HER and OER follow different pathways/mechanisms in various electrolytes. Currently, HER mechanisms have been well disclosed by experimental and computational investigations. Generally, HER obeys the Volmer/Tafel or Volmer/Heyrovsky routes. In alkaline media, there are four elementary steps (i.e., H_2_O adsorption, H_2_O dissociation, OH^−^ adsorption, and H_2_ generation) (Fig. 1b) [50]. Of note, the H_2_O adsorption and dissociation steps in alkaline HER show higher energy barriers than H_3_O^+^ adsorption in acidic HER. As a result, the activity of some catalysts (e.g., Pd, Pt) for acidic HER is theoretically much higher than that for alkaline HER [51]. It is suggested that HER catalysts with strong abilities to adsorb and dissociate H_2_O and bind protons would exhibit improved HER activities in alkaline media [52].

Different from the 2-electron HER process, the mechanism of the 4-electron OER is more complicated. Currently, the most acceptable OER pathways include the adsorbate evolution mechanism (AEM) and the lattice oxygen participation mechanism (LOM) [3]. As depicted in Fig. 1c, AEM for alkaline OER generally follows four steps. First, the oxidation of OH^−^ on the electrocatalytically active site (M) forms the intermediate M–OH. Then, the M–OH becomes M–O through a proton coupling-electron transfer process. The M–O further transforms into the M–OOH intermediate via an OH^−^ coupled with 1-electron oxidation and eventually initiates another proton-coupled electron transfer process to generate O_2_ molecules. Different from alkaline OER, the first step of AEM for acidic OER is the adsorption of a H_2_O molecule on M (Fig. 1d). Then, the dissociation of a H^+^ leads to the generation of M–OH, which is followed by the release of the second H^+^ to produce M–O. After that, M–OOH is formed after the nucleophilic attack of another H_2_O molecule. The final step is the desorption of the formed O_2_ molecule and the fourth proton coupling.

Recently, growing studies have focused on determining the origin of oxygen in O_2_ products, and some studies found that catalysts’ lattice oxygen participates in the OER process, namely the LOM-driven OER [53–55]. Taking LOM for alkaline OER as an illustration (Fig. 1e), OH^−^ is first adsorbed on the oxygen vacancy (O_v_)-coordinated active site (M–OH/O_v_). Subsequently, the O_v_ site near M adsorbs an additional OH^−^ and forms the M–OH/–OH species, which is followed by a dehydrogenation process and leads to the generation of M–OH/–O. Nevertheless, OH^−^ is difficult to undergo further dehydrogenation directly, and an unstable transition state (M–OH) is produced, which consequently transforms into M–OO/O_v_. At last, with the desorption of the formed O_2_ molecule and filling of OH^−^, the initial state M–OH/O_v_ is recovered. It is worth noting that the OER mechanism is highly sensitive to catalysts’ surface properties, and the in situ structural reconstruction of catalysts under OER conditions can regulate the catalysis process. To attain a better understanding of OER mechanisms, employing advanced techniques to investigate the structure self-evolution of catalysts and monitor the reaction intermediates (e.g., OH, OOH) is highly suggested [56].

### Parameters for Electrocatalyst Evaluation

Rational evaluating catalysts’ activities is important for advancing the design of high-performance electrocatalysts. Hence, several parameters have been proposed, including overpotential (*η*), Tafel slope, Faradaic efficiency, turnover frequency (TOF), and stability.

Overpotential (*η*) means the extra potential which is necessitated to initiate the electrochemical reactions. In general, *η* at a specified current density (*j*, e.g., 10 mA cm^−2^) is employed to assess the activity of electrocatalysts [57], and a lower *η* represents a higher activity.

Extracted from linear sweep voltammetry (LSV) curves, Tafel plots are employed to disclose the kinetics of electrochemical reactions [24]. The linear regions of Tafel plots can be fitted with the Tafel equation (*η* = *a* + *b*log *j*, where *b* represents the Tafel slope). When *η* is zero, the corresponding *j* obtained from the Tafel equation is termed the exchange current density (*j*_0_). *j*_0_ shows electrocatalysts’ intrinsic activity in the equilibrium state, which is generally used for HER catalysts’ evaluation.

Faradaic efficiency unveils the utilization efficiency of electrons involved in electrochemical reactions (i.e., HER, OER). Generally, Faradaic efficiency can be gained by comparing the experimental and theoretical values of gas product amounts. The amount/volume of gas products can be obtained via the internal water displacement method or tested with gas chromatography. Also, the fluorescence-based oxygen sensing method [58] and rotating ring disk electrode voltammetry [59] have been employed to measure the amount of oxygen gas.

TOF is explicated as the number of reactants (H_2_O) that electrocatalysts can convert to desired products (O_2_ or H_2_) per catalytic site per time unit. Accordingly, TOF demonstrates catalysts’ intrinsic activity. The value of TOF is generally calculated with the equation, TOF = (*jA*)/(*αFn*), where *j* is the current density at a fixed *η*; A means the surface area of the electrode; *α* is the electron numbers of the reaction; F represents the Faraday’s constant; *n* means the number of moles of the active sites. Of note, not all of the sites/atoms on catalysts are catalytically active or equally accessible, and thus it is difficult to gain an accurate TOF value for electrocatalysts. However, it is still rational to compare the TOF value of similar electrocatalysts.

Stability is a principal index that governs the practicability of electrocatalysts in commercial applications [60]. Two methods are generally used to test electrocatalysts’ stability. The first one is to document chronopotentiometry or chronoamperometry curves in a long-term running. The second one is the accelerated degradation test, which measures cyclic voltammetry (CV) or LSV curves for thousands of cycles. A stable catalyst would show an insignificant shift of potential or current density after the test.

Currently, it is still challenging to provide the best values of these parameters that are required for industrial water electrolysis applications of catalysts, because the measurement of these data is different from one study to another in terms of experimental protocols and catalysts’ properties (e.g., substrate, loading amount). Nevertheless, a promising electrocatalyst should possess a low *η*, a high Tafel slope, a high Faradaic efficiency, a high TOF, as well as good long-term stability.

### Design Principles of Efficient Catalysts for Water Electrolysis

**Fig. 2:**
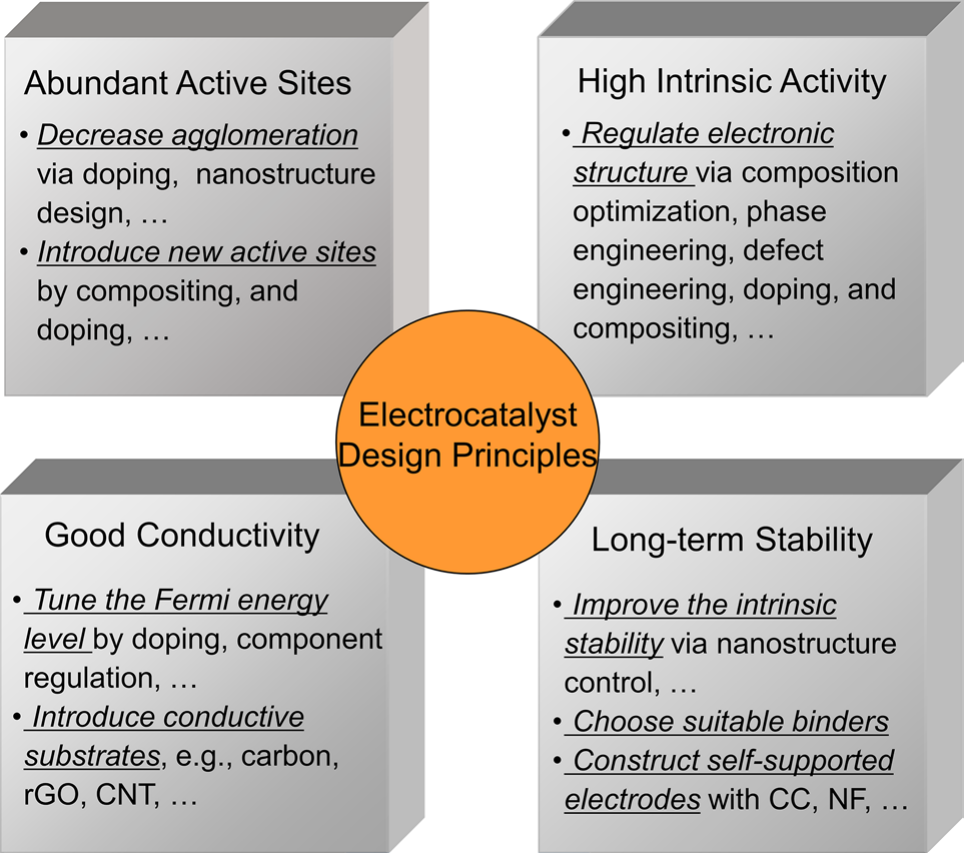
Design principles of waste-derived catalysts for water electrolysis

To develop high-performance electrocatalysts for water electrolysis, four general design principles should be kept in mind. As depicted in Fig. 2, abundant active sites, high intrinsic catalytic activity, good conductivity, and long-term performance durability/stability are essentials for a high-performance electrocatalyst. To attain these essential properties, diverse methods have been applied to regulate the internal and external characteristics of catalysts, such as doping, defect engineering, and nanostructure control. In this part, the most widely used methods for engineering efficient catalysts are detailed.

#### Abundant Electroactive Sites

Rich electroactive sites are necessary for the close contact of catalysts with electrolytes/reactants and promote electrocatalytic processes. Generally, there are four methods to construct abundant electroactive sites: (i) Dispersing catalyst particles on substrates with a high specific surface area (SSA) will reduce the agglomeration of catalysts and populate the electroactive sites; (ii) Reducing the size of catalysts by controlling the catalyst synthesis protocols allows the formation of nanosized catalyst particles. This method also can enhance the utilization efficiency of catalysts, a representative is single-atom catalysts; (iii) Previous studies also suggest that chemical doping and component regulation can tune the size of electrocatalysts and optimize the electroactive sites [61]; and (iv) Introducing electroactive dopants/materials can bring in additional catalytic sites, thus enriching surface electroactive sites.

#### High Intrinsic Catalytic Activity

Catalysts’ intrinsic catalytic activities largely dominate their electrocatalytic performance. Current strategies (e.g., composition optimization, heterostructure construction, doping, phase engineering, and defect engineering) for upgrading the catalysts’ intrinsic activity mainly focus on altering catalysts’ electronic structures. Typically, the *d*-band center and the density of state (DOS) are important electronic properties that provide meaningful information about the electron transfer behavior and reactant bonding/adsorption mechanisms on electroactive sites. Hence, applying apt strategies to modulate the electronic structure of catalysts is considered a powerful method to achieve suitable adsorption strengths/energies of reactants/intermediates (e.g., *H, *OH, *OOH) on electroactive sites, thereby high intrinsic activities.

#### High Electrical Conductivity

The electron transfer efficiency plays a crucial role in electrochemical reactions, and a high electrical conductivity can enhance electron transport throughout catalysts and prevent unwanted resistance at the electrolyte/catalyst interface [62]. In theory, the Fermi energy level of catalysts acts as the driving force of electron transfer [63], and better conductivity is associated with a higher electron density near the Fermi energy level. In this context, performing design strategies, like component regulation, cationic doping, and constructing heterostructures, on catalysts can attain a favorable Fermi energy level, and finally a high conductivity. Additionally, downsizing of catalyst particles and loading electroactive materials on conductive supports (e.g., nickel foam (NF), carbon papers (CP), reduced graphene oxides (rGO), and carbon nanotubes (CNT)) can also improve the electrical conductivity of entire catalysts/electrodes.

#### Long-term Performance Stability

To realize sustainable hydrogen fuel generation via water splitting, it is vital to maintain the performance stability of electrocatalysts in highly acidic or alkaline electrolytes. Electrochemical corrosion and detachment of electroactive materials are two major reasons for the degradation of electrodes. To overcome these barriers, component regulation, nanostructure control, and construction heterostructures can enhance the chemical and mechanical stability of catalysts under electrochemical conditions. Alternatively, developing electroactive materials on conductive and porous materials (e.g., NF, CP, porous carbon) via hydrothermal/solvothermal synthesis, electrodeposition, and electroless deposition can realize highly stable binder-free electrodes. For chemical binders-involved electrodes, the corrosion resistance property of binders to electrolytes also needs consideration, in addition to the stability of electroactive materials.

## Strategies for Converting Wastes to Catalysts

Pristine wastes can hardly be used as efficient catalysts for water electrolysis. To this end, converting diverse wastes (e.g., biowastes, industrial wastes) into high-performance catalysts is required. Of note, engineering electrocatalysts from wastes can significantly decrease the catalyst preparation cost as well as the negative impacts of wastes on the environment [42]. Waste-derived catalysts’ performance is largely determined by design principles that govern the nanostructure and surface chemistry of catalysts. In this part, mainstream principles for waste-derived catalyst design are discussed, including pyrolysis, electrochemical synthesis, wet-chemical methods, microwave synthesis, and others.

### Pyrolysis

Pyrolysis or carbonization is a frequently used process to design carbon-based electrocatalysts from biowastes [37, 64]. The pyrolysis/carbonization process is generally performed in a tube furnace under high temperatures, in an oxygen-free or oxygen-deficient atmosphere [65]. Catalytic properties of biowastes-derived electrocatalysts profoundly rely on parent biowastes’ properties (e.g., the ratio of heteroatoms, porous structure) and pyrolysis conditions (e.g., atmosphere, temperature, and time) [66]. Moreover, a general method to optimize the nanostructure/porosity is chemical activation during the pyrolysis process [67], and commonly used activators include KOH, K_2_CO_3_, ZnCl_2_, H_3_PO_4_, etc. Starting from peanut shells, Saravanan et al. developed multilayer carbon nanosheets for HER via a pyrolysis method. With KOH activation, the carbon material gains a high SSA (2338.5 m^2^ g^−1^) and uniform mesopores which improve the HER performance [68]. Aside from those one-step activation methods, several studies have proposed two-stage activation strategies to achieve a high surface area of carbon materials [69, 70]. For instance, Osman et al. used a two-stage H_3_PO_4_-KOH activation process to convert biowaste into carbon materials with a high surface of 1368 m^2^ g^−1^ and a pore volume of 0.92 cm^3^ g^−1^ [69].

**Fig. 3:**
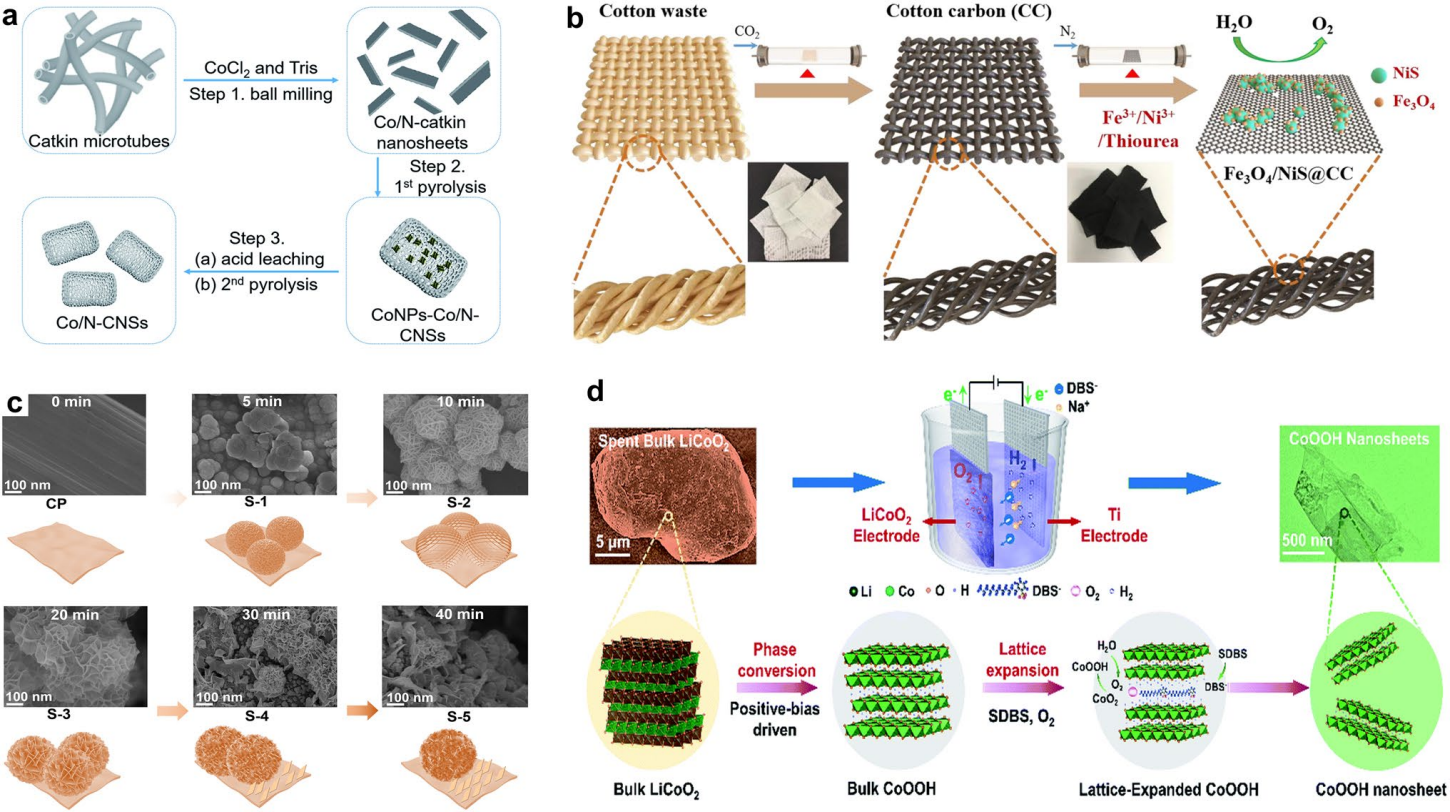
**a** Illustration of synthesis of Co/N–CNSs catalysts [73]. Copyright 2020, Royal Society of Chemistry. **b** Scheme of the preparation of Fe_3_O_4_/NiS@CC [74]. Copyright 2020, Elsevier. **c** Scanning electron microscopy (SEM) images and the scheme of NiCoMn LTHs catalysts nanostructure evolution during the electrodeposition process [39]. Copyright 2022, Royal Society of Chemistry. **d** Scheme of the positive-bias-driven exfoliation of LiCoO_2_ into CoOOH catalyst [79]. Copyright 2022, Royal Society of Chemistry

Besides using biowastes-derived materials as electrocatalysts directly, growing studies have designed carbon-based composites/heterostructures by pyrolysis [71]. The general process involves pyrolyzing the mixture of biowastes and metal salts, which could lead to the formation of metal compounds/carbon hybrids. For both HER and OER, the hybrids usually outperform the corresponding single components due to the populated electroactive sites and regulated electronic properties of metal compounds/carbon [72]. For instance, Song and co-authors designed Co and N co-doped carbon nanosheets (Co/N–CNSs) for HER from the catkins waste by a ball-milling two-stage pyrolysis process (Fig. 3a) [73]. The Co/N–CNSs show good HER activities on account of the formation of well-dispersed CoN_x_ sites on carbon structures. Another effective method to develop waste-derived carbon-based composites is functionalizing carbon materials with electroactive nanomaterials (e.g., phosphates, oxides, sulfides) by a post-treatment (e.g., hydrothermal method, carbonization) [74, 75]. As illustrated in Fig. 3b, the Fe_3_O_4_ and NiS hybrid nanoparticles are formed on the cotton carbon (CC) via a post-carbonization treatment under a N_2_ atmosphere. The as-obtained Fe_3_O_4_/NiS@CC catalyst displays good OER performance with a low overpotential (*η*_10_ = 310 mV), outperforming its counterparts [74].

### Electrochemical Synthesis

Electrochemical synthesis (e.g., electrodeposition) is powerful for engineering electrocatalysts from metal laden wastes or preparing electrocatalysts on low-cost robust substrates, such as spent stainless steel and cable wires [76–78]. The electrocatalysts’ properties are highly dependent on the property of wastes (electrolytes) and experimental protocols (e.g., deposition period, current, temperature, potential) since such parameters largely influence catalysts’ surface chemistry and nanostructure. Using the battery industrial wastewater as metal precursors, Chen et al. found that the nanostructure and elemental composition of electrodeposited NiCoMn-layered triple hydroxides (LTHs) electrodes obtained at different deposition periods were different (Fig. 3c). Specifically, the electrodeposit transforms from nanoclusters (S-1, 5 min) and nanospheres (S-2, 10 min) to nanoflowers (S-3, 20 min) and nanoplates (S-4, 30 min; S-5, 40 min). The optimal catalyst (S-3) featuring a hierarchical nanostructure, low crystallinity, and a high metal content of 67.33% possesses higher electrocatalytic activities toward both OER and HER [39].

Electrochemical transformation of solid metal-bearing wastes under a potential can also lead to high-performance electrocatalysts. Huang and coworkers developed a positive-bias-driven exfoliation method to convert spent LiCoO_2_ electrode materials into CoOOH which shows high OER performance (Fig. 3d) [79]. This electrochemical exfoliation process provides an eco-friendly, and high-efficiency route for constructing electrocatalysts by destroying the crystal structure of parent materials and oxidizing the electroactive elements to a high-valence state, which is suggested to benefit the OER process.

### Wet-Chemical Methods

**Fig. 4:**
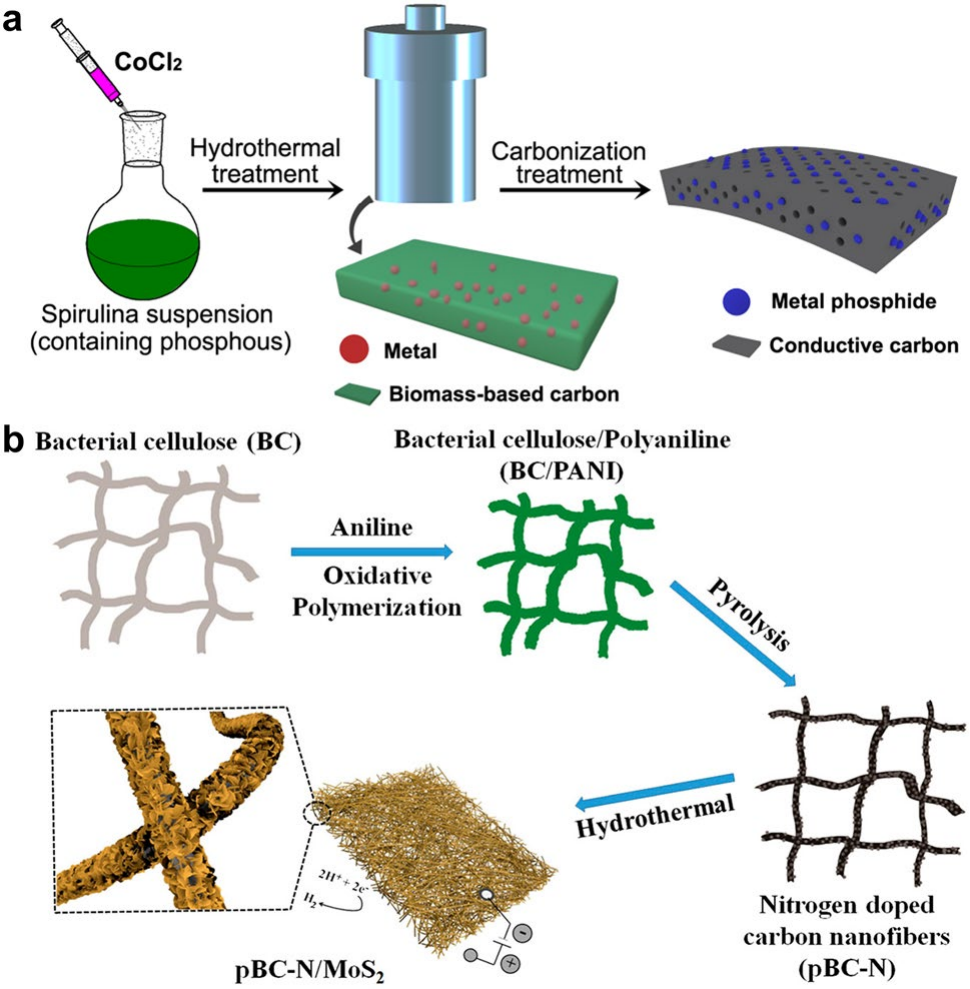
**a** Scheme of synthesis of cobalt phosphides decorated spirulina-derived porous N-doped carbon matrix (Co_2_P/NC) catalyst [88]. Copyright 2020, Wiley–VCH. **b** Illustration of the fabrication of N-doped carbon nanofiber/MoS_2_ (pBC-N/MoS_2_) nanocomposites [43]. Copyright 2016, American Chemical Society

Wet-chemical methods are widely used for preparing electrocatalysts from diverse wastes, including hydrothermal/solvothermal synthesis, sol–gel process, and boriding [80–82]. All these processes involve chemical reactions in solutions, with different temperatures, pressures, and chemicals. Among them, hydrothermal/solvothermal methods are the most frequent applied one to synthesize carbon [83], metal oxides/hydroxides/sulfides/phosphides, and heterostructural catalysts [84, 85], especially carbon-based composites, such as Co_2_P/C [86], MoS_2_/C [87], Co_3_O_4_/NHPC (nitrogen-doped hierarchically porous carbon) [40]. Two processes are generally involved to construct composite electrocatalysts from biowastes with the hydrothermal method. The first one is converting biowastes into carbon via hydrothermal carbonization and metal salts are also involved, which is followed by a post-treatment (e.g., pyrolysis) to form hybrid catalysts (Fig. 4a) [88]. The second method is developing electroactive metal species on the pre-synthesized biowastes-derived carbon by a hydrothermal process (Fig. 4b) [43].

The sol–gel method is generally combined with a thermochemical process to synthesize carbon-based heterostructures from biowastes. Starting from agarose biowastes, Xiao and co-authors proposed a sol–gel–calcination route to prepare Fe-Ni_2_P nanoparticles decorated N, P co-doped carbon catalyst (Fe-Ni_2_P@N, P-CNSs) [89]. Thanks to the enhanced electrical conductivity, high SSA, and rich electroactive sites, the Fe-Ni_2_P@N, P-CNSs catalyst shows high OER activities. Recently, researchers have developed a new boriding process to transform metal laden wastes into high-performance OER catalysts [90]. The boriding route refers to the reduction of metal species (e.g., Co, Ni, Cu, Fe, Mn, and Sn) in wastes and the generation of metal boride nanoparticles. The catalytic properties of obtained metal borides are governed by wastes’ properties (e.g., metals’ species and contents) and boriding protocols (e.g., atmosphere, temperature, reductants’ amount). In general, metal borides with small sizes, high dispersion, and a high ratio of Ni and Fe exhibit high OER performance [45].

### Microwave Synthesis and Beyond

Microwave-assisted synthesis is efficient for nanocatalysts preparation because of its unique merits of short reaction time, cleanliness, and high energy utilization efficiency [91]. More importantly, different from conventional heating strategies (e.g., hydrothermal process, calcination), the microwave-assisted heating process can realize uniform heating and facilitate crystal nucleus generation/crystallization rapidly [92]. In 2018, Cova et al. proposed a microwave-assisted strategy to design Ag/Ag_2_S-carbon hybrid from pig bristles. The pig bristles can be efficiently decomposed with microwave heating, and the discharge of S facilitates the formation of Ag_2_S [41]. More recently, Miao and co-authors employed a microwave hydrothermal route to construct a NiFe-borate layered double hydroxide/biomass-derived N-doped carbon (NiFe-BLDH/NC) hybrid catalyst [93]. With multistage decentralized architecture, rich active sites, good electrical conductivity, and efficient charge/mass transfer kinetics, the NiFe-BLDH/NC shows high OER activities (*η*_10_ = 243 mV, Tafel slope = 42.7 mV dec^−1^). Compared with microwave synthesis, Zuliani et al. suggested that ultrasound treatment was better for the synthesis of Co/pinecones-derived carbon hybrid OER catalysts. Further analysis indicates that the ultrasound method leads to a higher number of electroactive sites than the microwave, microwave/ultrasound, and conventional heating processes [94].

Apart from the aforementioned methods, biogenic synthesis also has been employed for preparing electrocatalysts from wastes [95]. Generally, the synthesis of electrocatalysts from wastes involves a combination of different methods, representatives include hydrothermal–pyrolysis, pyrolysis–hydrothermal, sol–gel–calcination/pyrolysis, and pyrolysis–microwave processes. The rich combinations of synthesis methods allow the construction of diverse high-performance catalysts for water electrolysis.

## Waste-derived Catalysts for HER

Developing cost-effective HER electrocatalysts allows sustainable and efficient hydrogen generation at the cathode part of water electrolyzers [96–98]. Currently, electroactive carbon materials, transitional metal-based catalysts, and carbon-based heterostructures synthesized from wastes have shown good HER performance in a wide pH range (Table 1). This part reviews recent advances in representative waste-derived HER electrocatalysts.

**Table 1:** Summary of representative waste-derived HER electrocatalysts

Waste	Catalyst	Electrolyte	*η*_10_ (mV)	Tafel slope (mV dec^−1^)	Refs
Palm plant	Hierarchical porous carbon nanosheets	0.5 M H_2_SO_4_	330	63	[109]
Human hair ashes	Partially graphitized activated carbon nanobundles	0.5 M H_2_SO_4_	16	51	[67]
Rice husks	Corrugated graphene nanosheets	0.5 M H_2_SO_4_	9	31	[99]
Paper wastes	Co, N co-doped carbon	0.5 M H_2_SO_4_	223	91	[112]
Waste tires	Zn, S, N co-doped carbon	1.0 M KOH	50	78	[111]
Waste cotton textile	O-doped biochar	0.5 M H_2_SO_4_	247.6	120.8	[196]
Polysaccharides	Defective N-doped graphene sponge	0.5 M H_2_SO_4_	267	69.7	[104]
Pistachio shells	Ni, N co-doped carbon	1.0 M KOH	403	146	[113]
Plastic wastes	Holey and wrinkled graphene	0.5 M H_2_SO_4_	613	91	[103]
Peanut shells	N-doped carbon nanosheets	0.5 M H_2_SO_4_	390	75.7	[68]
Tamarindus indica shells	Graphitic carbon	1.0 M KOH	221	204	[100]
Peanut root nodules	S, N co-doped carbon nanosheets	0.5 M H_2_SO_4_	27	67.8	[110]
Plastic wastes	N, O co-doped carbon	0.5 M H_2_SO_4_	309	87	[102]
Animal bones	N-, P- and Ca co-doped biochar	0.5 M H_2_SO_4_	162 ± 3	80	[115]
Cattail fibers	Porous N-doped carbon fibers	0.5 M H_2_SO_4_	248	135	[197]
Bean sprouts	N-doped carbon	0.5 M H_2_SO_4_	413	98	[38]
Plastic wastes	N-doped carbon coated Mo_2_C	0.5 M H_2_SO_4_	186.6	72.9	[124]
Plastic wastes	N-doped carbon supported MoS_2_	0.5 M H_2_SO_4_	56	36.6	[131]
Watermelon peels	Mo_2_C/C	1.0 M KOH	133	71	[198]
Waste-yeast cells	N, P co-doped Mo_2_C confined in porous carbon	1.0 M KOH	84	58.15	[126]
Electronic wastes	Au@N-doped carbon	0.5 M H_2_SO_4_	54.1	76.8	[123]
Aloe waste	ZnMoO_4_/carbon	1.0 M KOH	124	54	[129]
Fly ash	Fly ash/TiO_2_	0.1 M KOH	125	115	[120]
Banana waste	Pd/Fe_3_O_4_@carbon	0.5 M H_2_SO_4_	293	227.05	[199]
Wood residue	Mo_2_C	0.5 M H_2_SO_4_	35	25	[118]
Waste polythene	N-doped carbon-supported Mo_2_C	0.5 M H_2_SO_4_	197.37	69.2	[200]
Eggshell membrane	NiO/C	1.0 M KOH	670	77.8	[80]
Organic liquid waste	MoS_2_/vertical graphene nanosheets	0.5 M H_2_SO_4_	183	38	[132]
Ion-exchange resin	Cr_2_O_3_/C	0.5 M H_2_SO_4_	123	90	[128]
Coffee waste grounds	Carbon-coated Fe nanoparticles	0.5 M H_2_SO_4_	75	59	[122]
Carbon dioxide	Ni/NiO_x_@C	0.1 M KOH	337	–	[201]
Plastic waste	Mo_2_C/MnO_2_@C	0.5 M H_2_SO_4_	58.3	36	[46]
Walnut shells	Mo_2_C@C	0.5 M H_2_SO_4_	140	63	[125]
Sugarcane bagasse	Co-MoS_2_/C	0.5 M H_2_SO_4_	62	53.86	[133]
Chlorella	Co_2_P/N-doped carbon	0.5 M H_2_SO_4_	151	50.21	[202]
		1.0 M KOH	252	70.14	[202]
Silk cocoon	NiCo alloy/N-doped carbon	1.0 M KOH	179	40	[203]
Scrap nickel	Ni_2_P nanoparticles	0.5 M H_2_SO_4_	69	55	[119]
		1.0 M KOH	73	73	[119]
Pig bristles	Ag/Ag_2_S@C	0.5 M H_2_SO_4_	190	150	[41]

### Waste-derived Carbon Catalysts for HER

Carbon-based electrocatalysts exhibit some features for HER, including earth abundance, easily tunable nanostructure, and high stability in broad pH conditions. To date, the development of waste-derived carbon catalysts centrally focuses on phase regulation, nanostructure control, and heteroatom doping.

**Fig. 5:**
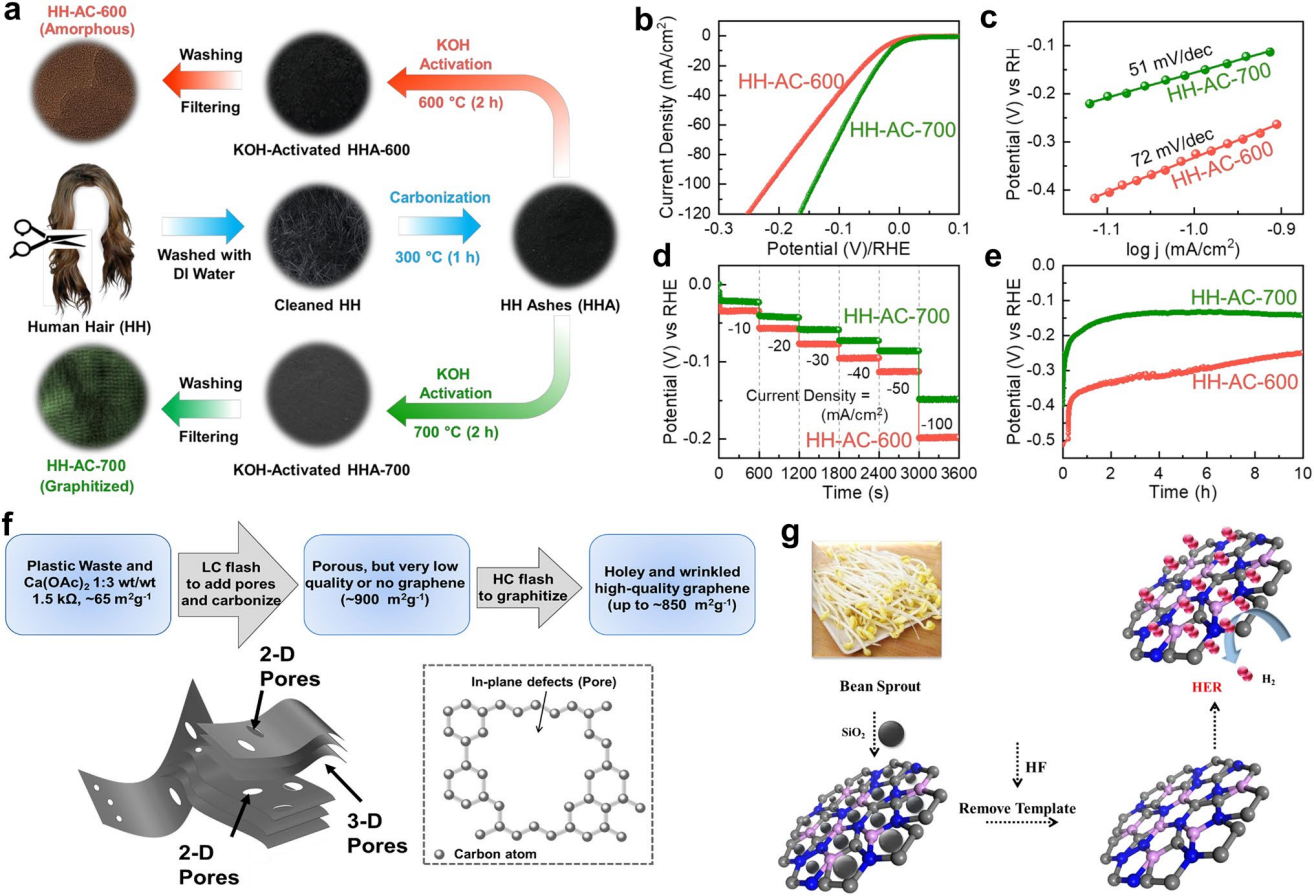
**a** Scheme of the preparation of human hair-derived HH-AC-700 layered nanobundles and HH-AC-600 nanobundles. **b** LSV curves, **c** Tafel plots, **d** multi-chronopotentiometry profiles, and **e** time-dependent HER stability for HH-AC-700 and HH-AC-600 catalysts [67]. Copyright 2022, MDPI. **f** Illustration of the HWFG preparation process, and the diagram of HWFG’s porous structure [103]. Copyright 2022, American Chemical Society. **g** Scheme of the fabrication process of bean sprouts carbon materials [38]. Copyright 2021, Elsevier

The phase/crystal structure of carbon catalysts influences their catalytic properties by determining the electrical conductivity, density of electroactive sits, and intrinsic catalytic activity. Starting from human hairs, Sekar et al. designed two carbon materials with different graphitization degrees (Fig. 5a) [67]. Compared with the amorphous carbon material (HH-AC-600) prepared at a lower temperature (600 °C), the catalyst synthesized at 700 °C (HH-AC-700) shows a partial graphitization feature. Interestingly, the HH-AC-700 catalyst possesses a higher textural porosity and higher electrical conductivity than its counterpart, which contributes to better HER activities in acidic media (Fig. 5b-c). Additionally, the HH-AC-700 catalyst exhibits better stability, as evidenced by the multiple chronopotentiometry and time-dependent measurements for 1 and 10 h, respectively (Fig. 5d-e). Similar results reported by the same group also indicate that rice husks-derived graphene nanosheets prepared at a higher temperature (700 °C) with a relatively higher crystallinity exhibit better HER activities [99]. However, not all reports follow this synthesis temperature-HER activity trend. Thirumal et al. found that the activated carbon catalyst obtained at 800 °C outperformed its analogues synthesized at 700 and 900 °C due to its highest conductivity [100].

Engineering the nanostructure of carbon catalysts is a powerful strategy to upgrade the HER performance. Carbon catalysts with diverse morphologies/nanostructures have been developed for HER, especially nanosheets and porous architecture. These structures feature large SSA, which contributes to efficient electrolyte percolation, abundant electroactive sites, and rapid mass/charge transfer during the catalytic process [101]. Some studies have emphasized the importance of managing porous structures in carbon catalysts. In the low-temperature solvothermal dehalogenation of polyvinyl chloride (PVC) wastes, the solid-base catalyst can work as a pore-forming additive to generate hierarchically porous carbon monolith [102]. More recently, Wyss and coworkers developed a Joule heating process to convert mixed plastic wastes into holey and wrinkled flash graphene (HWFG) (Fig. 5f) [103]. The obtained graphene contains rich three-dimensional (3D) and 2D pores and displays a large surface area (874 m^2^ g^–1^). Nevertheless, the HWFG only shows a mediocre HER activity (*η*_10_ = 613 mV) in an acidic solution, which may be due to its pure carbon composition. Using hard templates is another efficient method to engineer porous structures in carbon catalysts. Niu et al. designed graphene sponges by employing SiO_2_ spheres as the hard template and chitosan biomass as the carbon source [104]. With a spatial structure and high surface area, the obtained defective N-doped graphene sponge shows a good HER activity (*η*_0.5_ = 203 mV) and excellent durability for about 2 h. For potential applications, the stability test should be operated for a longer period to meet the industrial demand.

Doping is powerful to upgrade carbon catalysts’ intrinsic activities [105]. Compared with the nonpolar C–C bonds in pure carbon materials, carbon atoms in heteroatom-doped carbon materials can develop polar bonds with doped heteroatoms (e.g., N, P) to impose different dipole moments depending on their difference in electronegativity and atomic size from those of carbon [106]. Accordingly, an adjustment in the DOS and charge population can be achieved on both the carbon atom and heteroatoms, which would help to improve catalytic activities in various heteroatom-doped carbon materials [107, 108]. Biowastes themselves are effective sources for in situ synthesizing heteroatoms (especially N, S, O)-doped carbon materials [109]. For instance, Cao and coworkers utilized bean sprouts as the carbon precursor to prepare carbon materials due to their self-doping characteristics under the high-temperature calcination condition (Fig. 5g) [38]. The resulting N, S co-decorated carbon catalyst shows acceptable HER activities (*η*_10_ = 413 mV, Tafel slope = 98 mV dec^−1^) with high durability over 2000 CV cycles in acidic media. The influence of N and S dopants on HER performance was disclosed by density functional theory (DFT) calculations. Specifically, S dopants can lead to significant changes in the electronic structures and enhance the adsorption of the H atom intermediate on catalysts, which could improve the HER activity more efficiently than single N doping [110]. Besides nonmetal doping, transitional metals also have been incorporated into carbon materials, such as Zn, S, N self-doped carbon [111], Co, N co-doped carbon [112], Ni, N co-doped carbon [113, 114], and N, P, Ca co-doped biochar [115]. The presence of metal atoms can significantly improve catalytic performance by increasing the electrical conductivity and taking the advantage of synergistic effects of different elements [115]. A special structure is metal-N–C which emerges as a promising candidate for HER [114, 116]; as suggested, the abundant Co–N electroactive sites in the Co, N co-doped carbon (Co_x_–N–C) contribute to enhanced HER activities [112].

### Waste-derived Transitional Metal-based Catalysts for HER

Earth-abundant transitional metals, especially Fe, Cu, Ni, Co, and Mo, are extensively employed for designing high-performance HER catalysts due to their high conductivity, good electrochemical activity, as well as low cost [51, 117]. To further reduce catalysts’ fabrication cost, several studies have converted biowastes and industrial wastes into transitional metal-based HER catalysts.

**Fig. 6:**
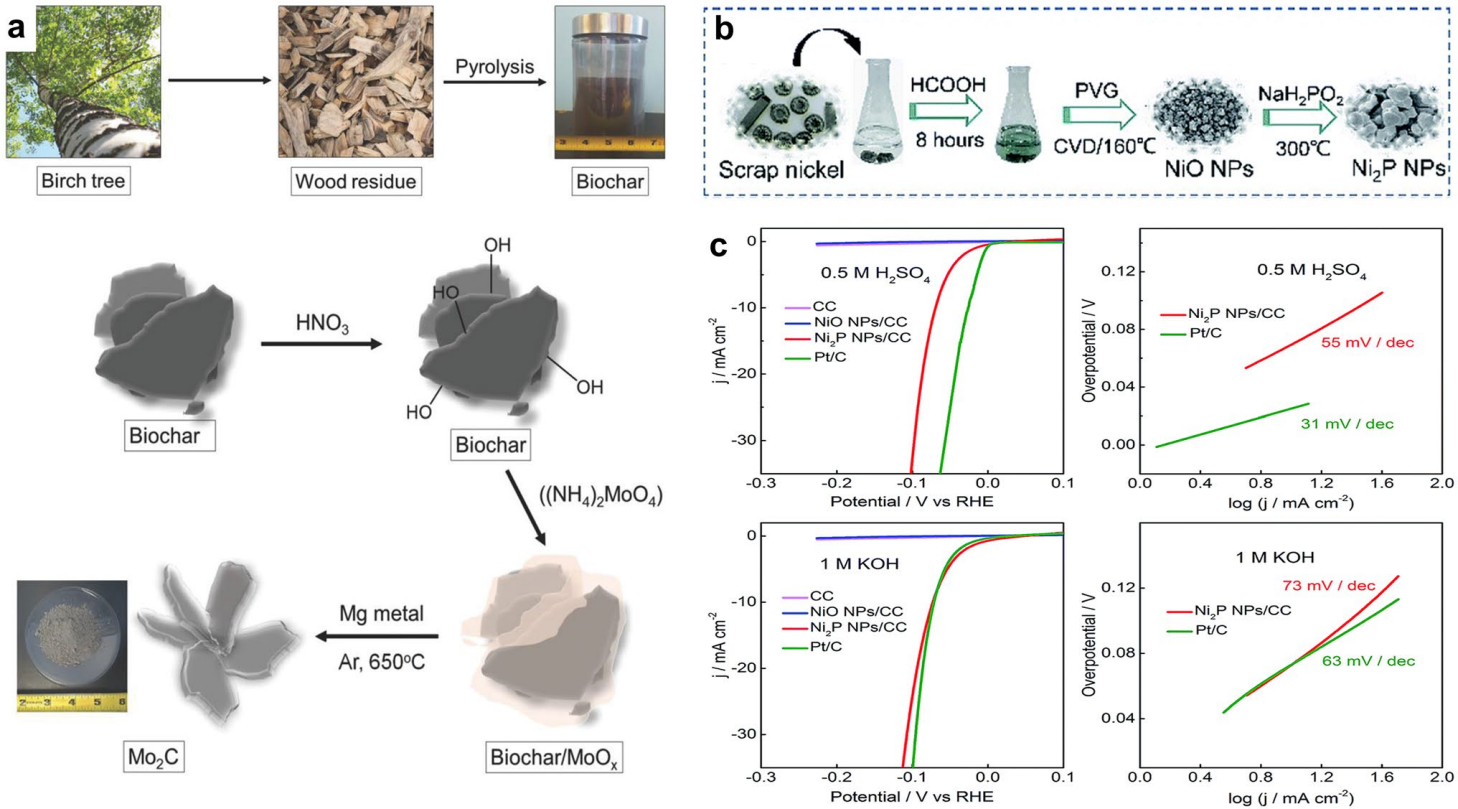
**a** Diagram of the preparation of the Mo_2_C catalyst from birch tree [118]. Copyright 2018, Wiley–VCH. **b** Scheme of the Ni_2_P catalyst preparation process (PVG: photochemical vapor generation; CVD: chemical vapor deposition). **c** HER performance of the Ni_2_P catalyst in acidic and alkaline media [119]. Copyright 2018, Royal Society of Chemistry

Starting from the birch tree, Humagain and co-authors design a porous Mo_2_C catalyst for HER (Fig. 6a), the biowaste-derived biochar acts as the carbon source instead of a carbon substrate [118]. The Mo_2_C catalyst can efficiently catalyze water reduction in the acidic electrolyte (*η*_10_ = 35 mV, *η*_100_ = 60 mV), with high durability for 100 h. Besides metal carbides, highly conductive metal phosphides also attain great interest. In 2018, Lin et al. reported a three-step process to transform bulk scrap nickel into 3D Ni_2_P nanoparticle catalysts (Fig. 6b) [119]. Benefiting from its high intrinsic activity and 3D nanostructure, the obtained Ni_2_P catalyst exhibits high HER activities in both alkaline and acidic electrolytes with low overpotentials of 73 and 69 mV at 10 mA cm^−2^, respectively (Fig. 6c-d). Compared with these single component transitional metal-based catalysts, constructing hybrids from wastes can realize enhanced HER performance. Altalhi and coworkers used industrial fly ash (FA) waste with TiO_2_ to create a FA-TiO_2_ nanocomposite [120]. With a post-cathodic polarization treatment, the activated FA-TiO_2_ nanocomposite catalyst shows good HER activities (*η*_10_ = 125 mV, Tafel slope = 115 mV dec^−1^) in the alkaline electrolyte, which are comparable to those of the Pt/C catalyst. Although the FA-based composite exhibits high electrocatalytic performance, it is challenging to identify the activity origin owing to the unclear crystal structure and complicated chemical composition of FA. Since most waste-derived transitional metal-based HER electrocatalysts also show high OER activities (e.g., NiCoMn hydroxides [39]), they will be discussed in the part of waste-derived bifunctional catalysts for OWE.

### Waste-derived Carbon-based Heterostructures for HER

Combining carbon materials’ large surface area and high conductivity and transitional metals’ high intrinsic activity can enhance the HER performance of individual components. In this regard, hybridizing biowaste-derived carbon with transitional metal-based nanomaterials is practical to create favorable HER electrocatalysts [121]. To date, a group of waste-derived carbon-based heterostructures has been realized for HER, such as metal–carbon and metal alloys/oxides/sulfides/phosphides/carbides-carbon hybrids.

**Fig. 7:**
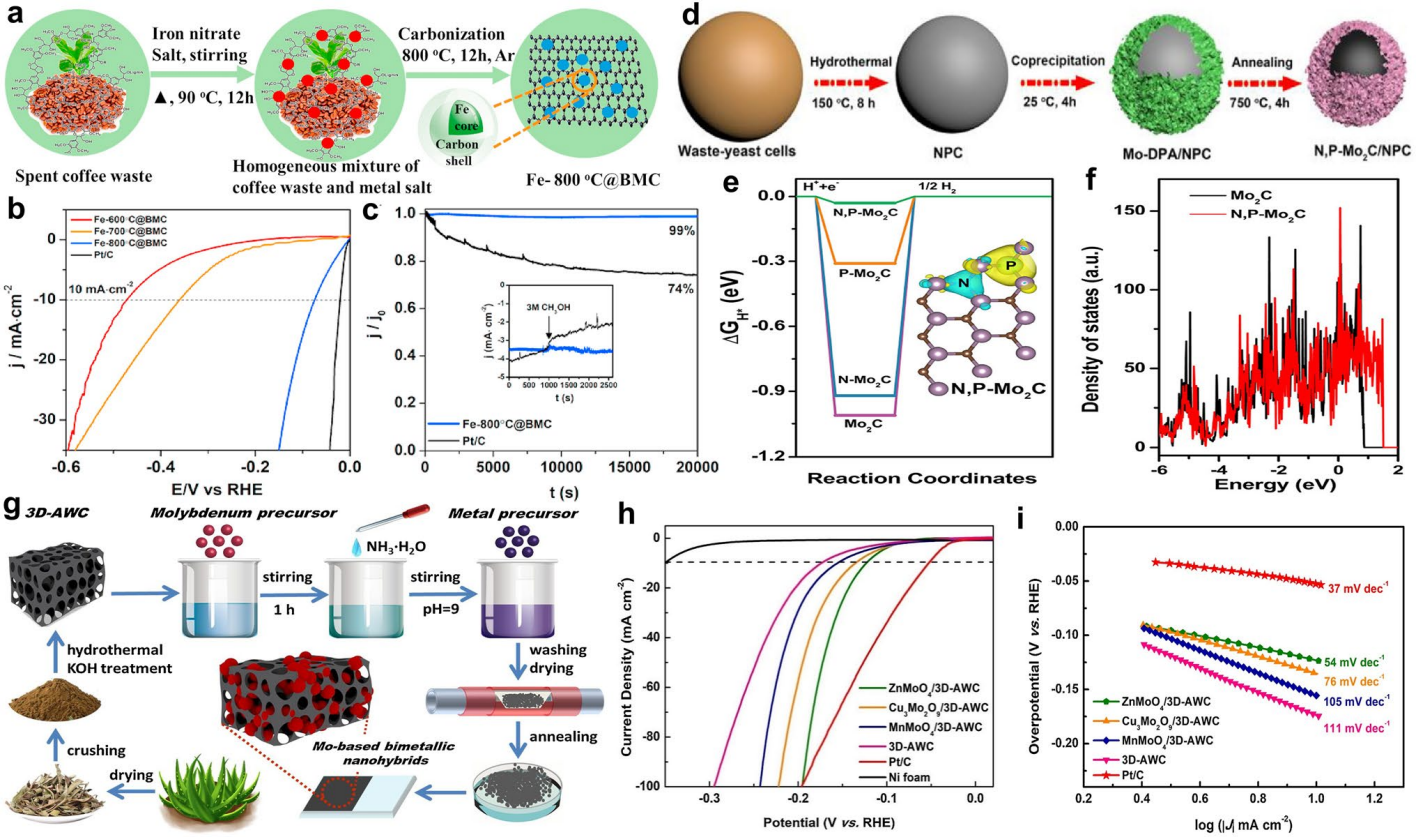
**a** Diagram of the preparation of Fe-800 °C@BMC catalyst. **b** LSV curves catalysts in 0.5 M H_2_SO_4_. **c** Chronoamperometric curves of Fe-800 °C@BMC and Pt/C catalysts at the overpotential of 350 mV, the inset shows the crossover effect of Fe-800 °C@BMC and Pt/C catalyst with the addition of 3 M methanol [122]. Copyright 2020, Elsevier. **d** Illustration of the reutilization of waste-yeast cells to design N, P-Mo_2_C/NPC catalyst. **e** Computational hydrogen adsorption free energy (ΔG_H*_) at the equilibrium potential, and the inset shows Bader charges of the N, P-Mo_2_C catalyst. **f** Calculated DOS for Mo_2_C and N,P-Mo_2_C catalysts [126]. Copyright 2022, Elsevier. **g** Scheme of the synthesis of Mo-based bimetallic oxides and their carbon-based hybrids. **h** LSV curves and **i** Tafel plots of catalysts in 1.0 M KOH [129]. Copyright 2020, Elsevier

Core@shell structured metallic particle@carbon catalysts with strong carbon–metal binding and high stability can be obtained by a reduction reaction. With a carbothermal reduction process, Ahsan et al. developed an ultrathin carbon-shell (4 nm)-coated metallic Fe nanoparticles structure (Fig. 7a) [122]. LSV measurements suggest that the sample prepared at 800 °C (Fe-800 °C@BMC) delivered high HER performance (*η*_10_ = 75 mV, Tafel slope = 59 mV dec^−1^) in acidic media, with high durability (99% of the initial activity preserved after 20000 s) (Fig. 7b-c). Both the hierarchically porous carbon matrix and the strong electronic interaction between carbon shells and metallic Fe cores contribute to the high catalytic performance. An earlier study reported a bio-reduction and calcination route to engineer Au nanoparticles covered by N-doped carbon (Au@NC) [123]. The interface interaction and charge transport between N-doped carbon and Au core significantly benefits the HER process and leads to high activities (*η*_10_ = 54.1 mV, Tafel slope = 76.8 mV dec^−1^).

Among all waste-derived carbon-based heterostructures, Mo_2_C/C catalysts are the most frequently studied. Traditionally, the synthesis of Mo_2_C needs a high temperature, which would result in severe agglomeration of particles [124]. Alternatively, encapsulating/loading Mo_2_C nanoparticles in/on a large surface carbon structure can enhance both catalytic efficiency and durability. Thus, biowastes have been extensively used to fabricate Mo_2_C/C catalysts [46, 125]. To further improve the catalytic performance, heteroatom doping is a favorable option. As shown in Fig. 7d, the N, P co-decorated Mo_2_C enclosed in the N, P co-doped carbon matrix (N, P-Mo_2_C/NPC) was prepared from waste-yeast cells [126]. The N, P-Mo_2_C/NPC hybrid displays a good HER activity (*η*_10_ = 84 mV) and high durability in alkaline solution. Further DFT calculations suggest that N and P dopants can significantly tune the electron density of electroactive sites on Mo_2_C and thus regulates the DOS of Mo_2_C, resulting in optimized intermediates adsorption energy (Fig. 7e, f). In the highly porous Mo_2_C/N-rich carbon matrix composite, the N dopant in carbon is also suggested to optimize the intrinsic activity by optimizing hydrogen adsorption strength [127].

Metal oxides/carbon heterostructures also attract enormous attention. Zhou et al. have tested the HER performance of different metal oxides/carbon catalysts prepared from spent ion-exchange resins [128]. Compared with other metal ions (i.e., Ni^2+^, Ag^+^, Pb^2+^, Mn^2+^, Cr^3+^, Cd^2+^, Zn^2+^ and Co^2+^), the Fe^3+^-contaminated resin-derived FeO_x_/C catalyst shows the best HER activity (*η*_10_ = 60 mV). Of note, the CrO_x_/C prepared from highly toxic metal Cr-containing ion-exchange resins can also attain a good catalytic activity (*η*_10_ = 123 mV), which provides a suitable route to reutilize hazardous wastes. Incorporating a second metal into metal oxides can enhance the catalytic performance. In some aloe waste-derived 3D carbon (3D-AWC)-supported Mo-based bimetallic oxides (ZnMoO_4_, MnMoO_4_, and Cu_3_Mo_2_O_9_) fabricated through a chemical precipitation route (Fig. 7g), the ZnMoO_4_/3D-AWC catalyst demonstrates a high HER activity in alkaline media (*η*_10_ = 124 mV, Tafel slope = 54 mV dec^−1^) (Fig. 7h, i) [129]. In-depth computational results indicate the good HER performance of Mo-based bimetallic oxides arises from metallic features and apt energy levels. Another efficient method to upgrade the HER performance of metal oxides/C hybrids is forming heterostructures on the carbon substrate. Upadhyay et al. have engineered a three-component Mo_2_C/MnO_2_/C heterostructure from laboratory plastic wastes [46]. In contrast to the Mo_2_C/C catalyst, the Mo_2_C/MnO_2_/C composite performs better toward HER. This is because the extremely fine and intertwine MnO_2_ nanoflakes develop a network that guarantees efficient electrons/ions transfer and enhances the structural stability over 5000 cycles of CV tests.

A group of metal sulfides/carbon hybrids also have been developed for HER recently. MoS_2_ is a representative HER catalyst among transitional metal dichalcogenides, due to its layer structures and abundant highly active sites [130]. Zhao et al. reported a plastic waste-derived carbon-supported MoS_2_ catalyst for HER [131]. The highly active MoS_2_ nanosheets are finely scattered on the carbon material. Interestingly, the rich pyridinic‒N in the carbon support provides additional electroactive sites, and there is a positive correlation between HER performance and the content of N dopant. The critical role of the carbon support in enhancing MoS_2_ catalysts’ HER performance is also identified in the organic liquid waste-derived vertical graphene nanosheets (VGNS)/MoS_2_ hybrid [132]. By combining VGNS with MoS_2_, the Schottky barrier height is reduced from 0.52 to 0.23 eV in the computational model, which is in line with the experimentally reduced overpotential by ∼ 50 mV. More recently, Ji and co-authors found that Co-doping could raise the HER performance of biowaste-derived MoS_2_/C [133]. The Co dopant can modulate the electronic structure of MoS_2_ and contribute to larger planar defect structures, which jointly ameliorate the HER performance of MoS_2_.

## Waste-derived Catalysts for OER

As a central bottleneck of the water electrolysis system, OER with inherently sluggish kinetics requires efficient electrocatalysts to speed the catalytic process [134]. Currently, cost-effective catalysts derived from diverse wastes (e.g., biomass, spent batteries) play a key role in upgrading OER performance. Similar to HER catalysts, the waste-derived OER catalysts listed in Table 2 also can be classified into three categories, namely carbon catalysts, transitional metal-based catalysts, and carbon-based heterostructures. It can be seen that most waste-derived OER catalysts only work in alkaline media because they are likely to be corroded, dissolved, and deactivated in harsh acidic and oxidative conditions [56, 135]. In this part, OER electrocatalysts synthesized from wastes are fully discussed, and some effective catalyst design strategies are outlined.

**Table 2:** Summary of representative waste-derived OER electrocatalysts

Waste	Catalyst	Electrolyte	*η*_10_ (mV)	Tafel slope (mV dec^−1^)	Refs
Lignocellulosic biowastes	N, P co-doped biochar	0.1 M KOH	347	28.8	[139]
Polymer waste	N-doped carbon	0.1 M KOH	497	–	[137]
		1.0 M HClO_4_	268	–	[137]
Plant residues	N-doped carbon	0.1 M KOH	450	–	[204]
Cedar tree cones	N-doped carbon	1.0 M KOH	106	191	[205]
Plant leaves	N-doped carbon	0.1 M KOH	340 (*η*_5_)	191	[138]
Corn stalks	Fe, N co-doped carbon nanosheets	0.1 M KOH	309	127	[141]
Cornstalks	Co, Fe, B, N co-doped biochar	1.0 M KOH	383	100.92	[140]
Waste printed circuit boards	FeNiCuSnB	1.0 M KOH	199	53.98	[45]
Spent Li-ion batteries	Ni_0.5_Mn_0.3_Co_0.2_(OH)_2_	1.0 M KOH	280	6.79	[157]
Stainless steel waste meshes	Anodized stainless steel	1.0 M KOH	280	63	[144]
Spent capacitors	FeNi hydroxides	1.0 M KOH	303 (*η*_20_)	80	[156]
Spent Li-ion batteries	Lithium cobaltate	1.0 M KOH	550	128	[148]
Waste steel alloy	Steel alloy	1.0 M KOH	387	64	[143]
Waste Cu cable wires	NiFe LDH^a^/Cu(OH)_2_/Cu	1.0 M KOH	275 (*η*_20_)	83	[78]
Spent Li-ion batteries	NiCoMnB	1.0 M KOH	263	57.98	[150]
Waste steel	Fe sheets	1.0 M KOH	439	60	[206]
Spent Li-ion batteries	MnCo_2_O_4_	1.0 M KOH	400	80	[146]
Spent Zn − C batteries	Mn_3_O_4_	0.1 M KOH	360	64	[207]
Spent Li-ion batteries	De-lithiated Li_0.4_Ni_0.5_Co_0.2_Mn_0.3_O_2_	1.0 M KOH	236	66	[149]
Spent Li-ion batteries	Ni-incorporated LiFePO_4_	1.0 M KOH	285	45	[76]
Rusty stainless steel	Activated stainless steel plate	1.0 M KOH	260	32	[145]
Spent Li-ion batteries	LiCoO_x_	0.1 M KOH	420 (*η*_9.68_)	67.41	[147]
Spent Li-ion batteries	CoOOH nanosheets	1.0 M KOH	301	53.8	[79]
Sludge waste	ZnS/N, S co-doped carbon	0.1 M KOH	390	117	[166]
Spent catalysts	Ni/CNTs/Al_2_O_3_	1.0 M KOH	370	119	[160]
Biowaste	Co/N-doped carbon	0.1 M KOH	390	72	[159]
Spent adsorbents	NiCuFeB/C	1.0 M KOH	251	71.75	[90]
Cotton fabric	Fe_3_O_4_/NiS@C	1.0 M KOH	310	82	[74]
Food waste	FeO_x_/nanocarbon	0.1 M KOH	~ 400	41	[162]
Peanut shells	FeNi alloy/N-doped carbon	0.1 M KOH	380	115	[161]
Cocoons	FeCoNi alloy/B, N co-doped carbon	1.0 M KOH	321	42	[158]
Diaper waste	NiO/C	1.0 M KOH	280	62	[208]
Spent Li-ion batteries	NiMnCo-activated carbon	1.0 M KOH	350	-	[165]
Waste paper	NiCo phosphate/C	1.0 M KOH	351	94.44	[75]
Onion peels	Fe_3_C@N-doped carbon	1.0 M KOH	330	52	[209]
Banana peels	Ba_0.5_Sr_0.5_Co_0.8_Fe_0.2_O_3−δ_/N-doped carbon	0.1 M KOH	350	65	[210]
Mangosteen skin	NiFe-borate LDH/N-doped carbon	1.0 M KOH	243	42.7	[93]
Milk powder	NiFeO_x_/N, P co-doped carbon	1.0 M KOH	320	59.03	[163]
Millet	Co_5.47_ N/N-doped carbon	0.1 M KOH	390	110	[211]
Polyphenylene sulfide	Fe, N, S co-doped carbon	0.1 M KOH	339	85.9	[169]
Red blood cells	CoS_1.097_/C	1.0 M KOH	260	83	[167]
Blood powder	Co_3_O_4_/C	0.1 M KOH	380	47	[164]
Spent Li-ion batteries	Co_3_O_4_/C	1.0 M KOH	245	53	[212]
Human hair	NiO/C	1.0 M KOH	320	49	[213]
Sludge	NiFe phosphide/heteroatom-doped carbon	1.0 M KOH	280	56	[214]

^a^ LDH: Layered double hydroxides

### Waste-derived Carbon Catalysts for OER

**Fig. 8:**
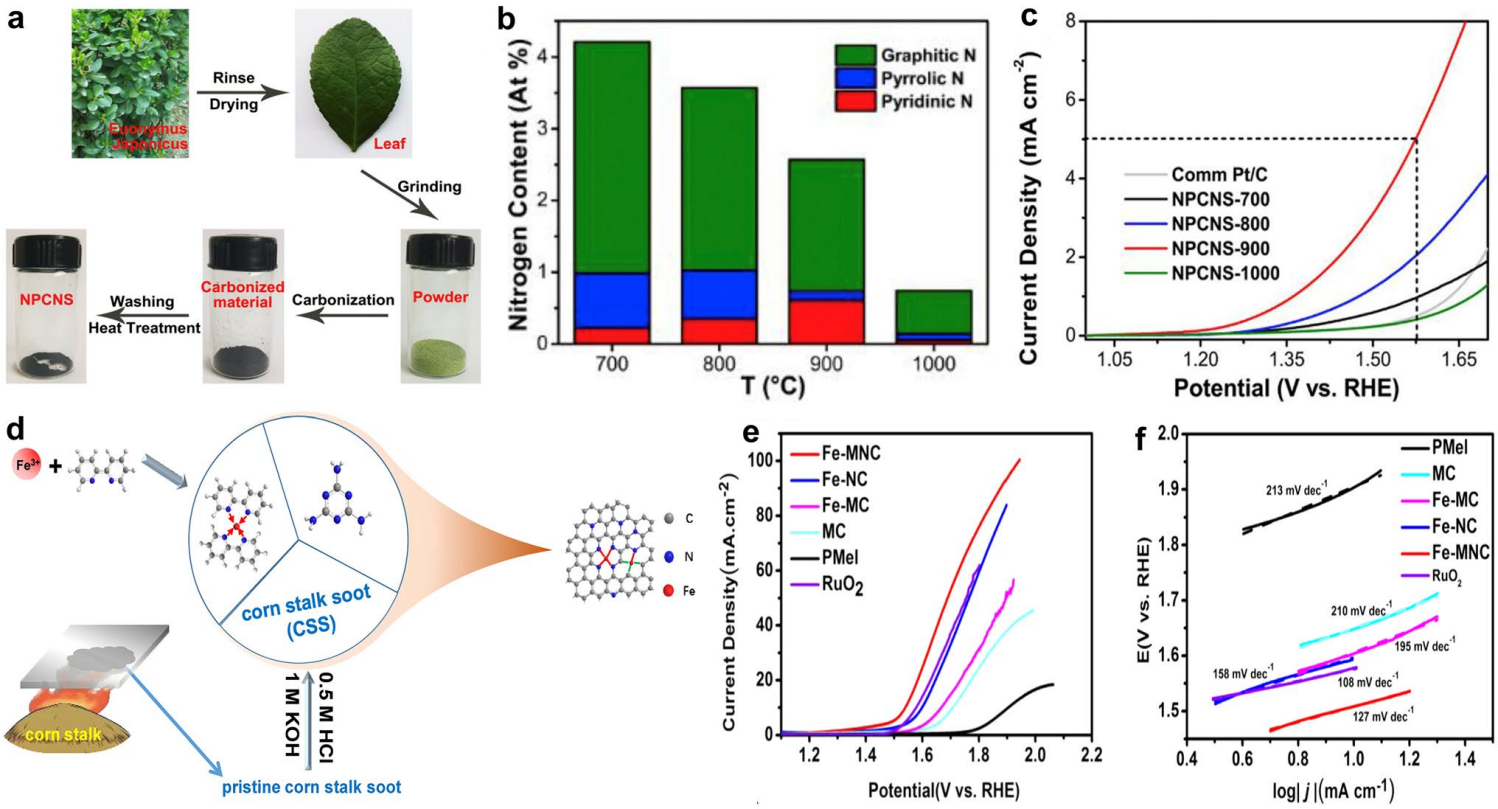
**a** Scheme of synthesis of N-doped porous carbon from plant leaves. **b** Nitrogen contents in carbon catalysts. **c** LSV curves of carbon catalysts and the Pt/C catalyst [138]. Copyright 2018, Elsevier. **d** Diagram of the fabrication of Fe, N co-doped mesoporous and microporous carbon (Fe-MNC). **e** LSV curves and **f** corresponding Tafel plots of catalysts in 0.1 M KOH [141]. Copyright 2022, Elsevier

Nanocarbon materials prepared from biomass and plastic wastes have shown promising OER performance, and most of them are N-doped carbon [136]. The benefit of N doping includes enhanced electrical conductivity, regulated surface electronic properties, increased structural disorder, and populated defective sites [137]. It is well accepted that the species of N dopants governs the catalytic activity of carbon catalysts. For example, the biomass (*euonymus japonicus* leaves)-derived N-doped porous carbon nanosheets (NPCNS) synthesized at different pyrolysis temperatures show distinct N contents (Fig. 8a, b) [138]. The sample obtained at 900 °C (NPCNS-900) contains the highest ratio of pyridinic-N, which contributes to its best OER performance compared to its analogues (Fig. 8c). The pyridinic-N shows more moderate adsorption energies toward O and OH intermediates than other N species (graphitic-N, pyrrolic-N), which is the most vital factor for the efficient OER performance of NPCNS-900.

Introducing another electroactive element into the N-doped carbon can effectively enhance the OER performance. Ma and co-authors developed a N, P co-decorated carbon catalyst from lignocellulosic biowastes [139]. Compared with the individual N- or P-doped carbon, the N, P co-doped carbon catalysts show a better OER activity. The main reason is that N and P co-doping contributes to favorable electronic structure and a variety of electroactive defect sites. Apart from nonmetal components, designing metal-N–C structures for OER also have been realized [140]. In Luo and coworkers’ study, the Fe, N co-doped porous carbon (Fe-MNC) catalyst obtained from corn stalk soot (CSS) (Fig. 8d) can catalyze water oxidation efficiently (*η*_10_ = 309 mV) [141]. This study also investigated the importance of chemical precursors on the nanostructure and electronic properties of catalysts. Compared with Fe-NC (sample without the presence of melamine), Fe-MC (sample without the presence of 2,2-dipyridine), MC (pyrolysis the hybrid of CSS and melamine), and PMel (pyrolysis bare melamine) catalysts, Fe-MNC with the optimal porous lamellar structure, less defects, and high concentration of active Fe-N_x_ and Fe-C_x_ sites exhibits better OER performance (Fig. 8e, f).

### Waste-Derived Transitional Metal-based Catalysts for OER

Transitional metal-based nanomaterials are efficient catalysts for alkaline OER [142]. Thus, many metal-rich industrial wastes have been employed to develop OER catalysts. Rich in Fe, ubiquitous steel wastes are great precursors for OER electrocatalysts. Maruthapandian et al. developed an OER electrocatalyst from high speed steel alloy by mechanical milling [143]. With major content of Fe, the steel alloy powder catalyst can act as a good pre-electrocatalyst for OER. After 50 h of the OER durability test, the waste-derived catalyst shows comparable OER activities to the RuO_2_ catalyst due to the formation of active metal (oxy)hydroxide phases on the catalyst surface. Starting from the industrial stainless steel 316L waste meshes, Gomaa and co-authors designed a self-supported OER catalyst (ASS-O_2_) via an anodization-annealing (O_2_ atmosphere) process [144]. Compared with catalysts prepared under other annealing atmospheres (i.e., H_2_, air), the ASS-O_2_ catalyst exhibits better OER activities owing to the formation of electroactive Fe_2_O_3_ with small amounts of FeCr alloy and NiO on the surface. A similar study also suggests that rusty stainless steel can be used as efficient free-standing OER catalysts due to the high conductivity, good mechanical stability, and especially the generated plentiful Fe/(Ni) oxyhydroxides on the catalyst surface during the electrochemical process [145].

**Fig. 9:**
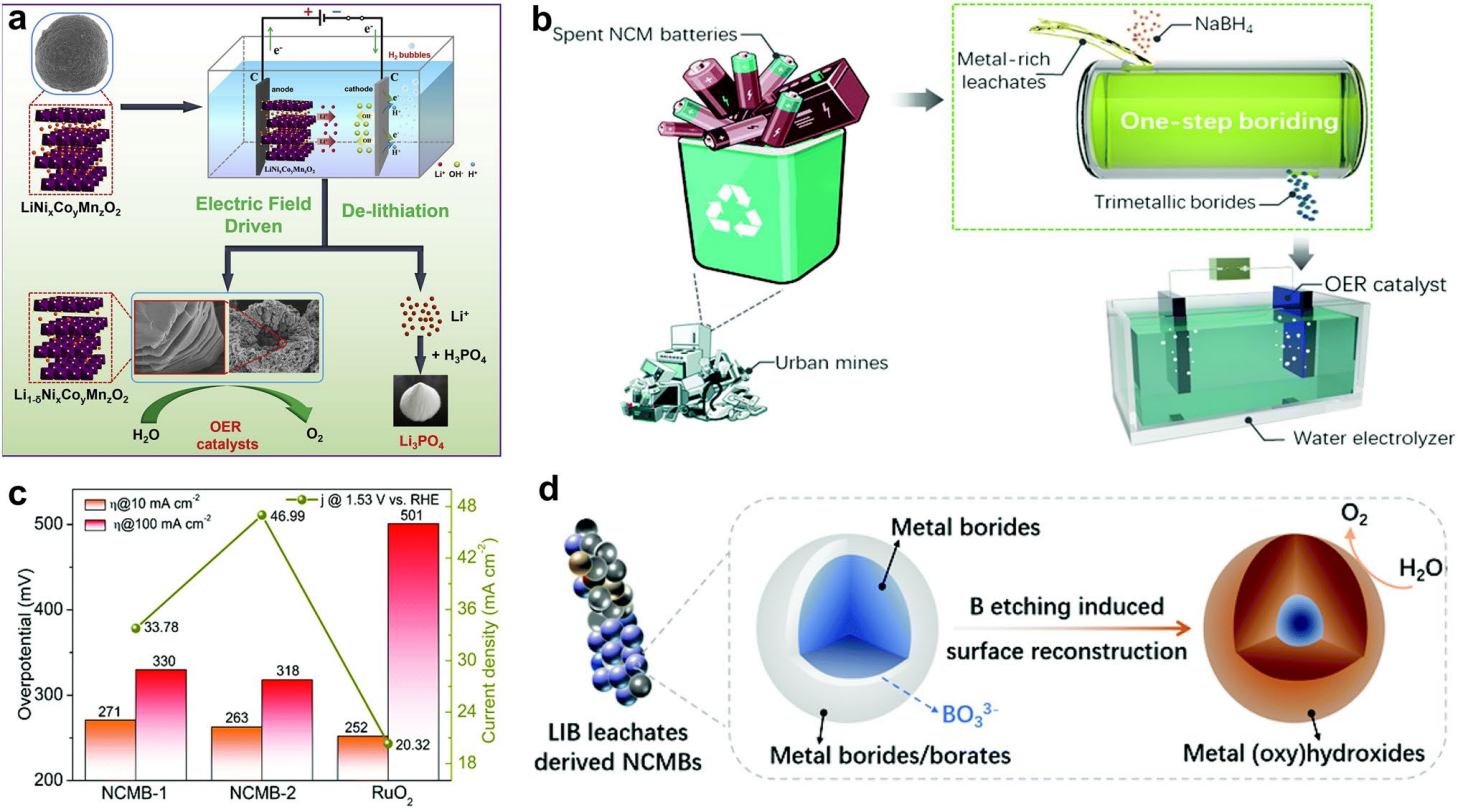
**a** Scheme of the electric field-driven de-lithiation process for preparing LiNi_x_Co_y_Mn_z_O_2_ OER catalysts [149]. Copyright 2021, Elsevier. **b** Illustration of the fabrication of magnetic NiCoMnB catalysts (NCMBs) from the spent batteries. **c** *η*_10_, *η*_100_, and the current density at 1.53 V (vs. reversible hydrogen electrode, RHE) of catalysts. **d** Scheme of the surface reconstruction process of spent Li-ion batteries-derived NCMBs [150]. Copyright 2021, Royal Society of Chemistry

Transitional metal oxides derived from spent batteries are a group of promising OER catalysts, and the chemical composition, surface chemistry, and nanostructure of oxides largely influence the catalytic properties. Natarajan et al. found that the OER performance of spent Li-ion batteries-derived spherical and porous spinel MnCo_2_O_4_ was better than the monometallic Co_3_O_4_ and MnO_2_ [146], and the reason for the better performance of MnCo_2_O_4_ has been attributed to its structural features. Lithium cobalt oxides can be easily obtained from Li-ion batteries. Chen and co-authors found that a long-time cycling treatment of LiCoO_x_ could result in smaller particle size and activated surface, and thus contributes to enhanced OER activities [147]. Another study introduced a solvent extraction-calcination method to recover LiCoO_x_ from Li-ion batteries [148]. The obtained oxides catalyst (calcinated at a low temperature of 600 °C) with optimal small particle size (20–100 nm) and surface area (4.8027 m^2^ g^−1^) outperforms its counterparts for OER. The synchronous reutilization of multi-metals in spent Li-ion batteries can not only shorten waste recycling procedures but also innovate mixed metal oxide catalysts. Lv et al. proposed an electric field-driven de-lithiation method to design high-performance OER catalysts from the cathode (Fig. 9a) [149]. The de-lithiated Li_0.4_Ni_0.5_Co_0.2_Mn_0.3_O_2_ cathode materials display a high specific surface area and a large amount of lattice oxygen, which contribute to high OER activities (*η*_10_ = 236 mV, Tafel slope = 66 mV dec^−1^). Sometimes, incorporating a foreign active species is necessary to enhance the catalytic properties of spent cathodes. For example, introducing a Ni promoter significantly improves the catalytic performance of spent LiFePO_4_ [76]. Theoretically, the insertion of Ni can effectively activate Fe sites by regulating the adsorption strength of the *OOH intermediate; also, the abundant oxygen defects promote the oxygen desorption step, which synergistically upgrade the spent LiFePO_4_ material’s OER performance.

Besides oxides, mixed metal borides synthesized from electronic wastes also have shown excellent OER catalysts [45]. As illustrated in Fig. 9b, spent Li-ion batteries can be directly converted into magnetic Ni-Co-Mn borides (NCMBs) through a fast and efficient NaBH_4_-mediated boriding process [150]. After the boriding reaction, the metal ion concentrations in the solution are below the emission limits of related standards, indicating the boriding process can ease the following waste effluent management process. The NCMB-2 catalyst with a larger ratio of Ni and Co content (38.4% vs. 20.3%) shows a higher OER activity (Fig. 9c), compared with the NCMB-1 analogue. In such a manner, it would be efficient to improve NCMBs’ OER performance by adjusting the composition of spent batteries precursors. In addition, the metal borides undergo surface reconstruction initiated by boron leaching and form stable metal (oxy)hydroxides on the catalyst surface (Fig. 9d). Such in situ surface/structure reconstruction processes have well been identified for various transitional metal-based catalysts [151–155], which becomes an important guideline for novel OER catalyst design.

The aforementioned studies emphasize the importance of metal (oxy)hydroxides because of their high catalytic performance and durability for alkaline OER. Consequently, it is sensible to develop metal (oxy)hydroxides directly from wastes. The NiFe hydroxides and NiCu hydroxides synthesized from upcycled capacitors [156], spent Li-ion batteries-derived Ni_0.5_Mn_0.3_Co_0.2_(OH)_2_ [157], NiFe LDH/Cu(OH)_2_/Cu prepared from spent Cu cable wires [78], and CoOOH obtained from spent Li-ion batteries [79] are representative efficient OER catalysts. Among these catalysts, the self-supported NiFe LDH/Cu(OH)_2_/Cu catalyst delivers a good OER activity (*η*_100_ = 390 mV) with excellent stability for 24 h, owing to its hierarchically heterostructural feature [78]. The multiphase heterostructure can ensure wealthy and multiple electroactive sites and endow fast mass/charge transport during OER.

### Waste-Derived Carbon-based Heterostructures for OER

The high conductivity, large surface area, and redox properties of carbon materials make them good substrates to support transitional metal-based electroactive nanomaterials, intending to achieve high OER performance. Different categories of transitional metal-based materials/carbon heterostructures have been synthesized from a range of wastes, which are detailed in this part.

Metal/alloy nanoparticles feature high electrical conductivity and catalytic activities. Compositing metal/alloy with carbon is capable of mitigating the severe aggregation and growth of metal/alloy nanoparticles, thus populating electroactive sites and also enhancing the structural stability of catalysts [158]. Chen et al. prepared a hierarchically structured catalyst (Co@Co–N, S–C) from biowaste, integrating Co–N–C structures and encased Co nanoparticles [159]. The well-developed interface between hierarchical structures, the large SSA, and rich Co nanoparticles encapsulated in carbon layers jointly contribute to a high OER activity. With suitable structural and electronic properties, the spent methane decomposition catalyst (Ni/CNTs/Al_2_O_3_) can be directly used as an OER catalyst [160]. In this tricomponent hybrid, Ni nanoparticles act as electroactive sites toward OER, CNTs can facilitate low charge transfer resistance, and the Al_2_O_3_ provides porous support. Although the authors declared good stability of the Ni/CNTs/Al_2_O_3_ catalyst for 20 h, it should be cautious that the Al_2_O_3_ support may suffer from leaching/dissolution in the strong alkaline electrolyte. Besides metal particles, Yang and coworkers developed a FeNi alloy/N-doped porous carbon catalyst from peanut shells [161]. The alloy/carbon hybrid prepared at 900 °C with a higher SSA and porous size outperforms its analogues for OER owing to the enhanced mass/charge transfer and abundant active sites.

**Fig. 10:**
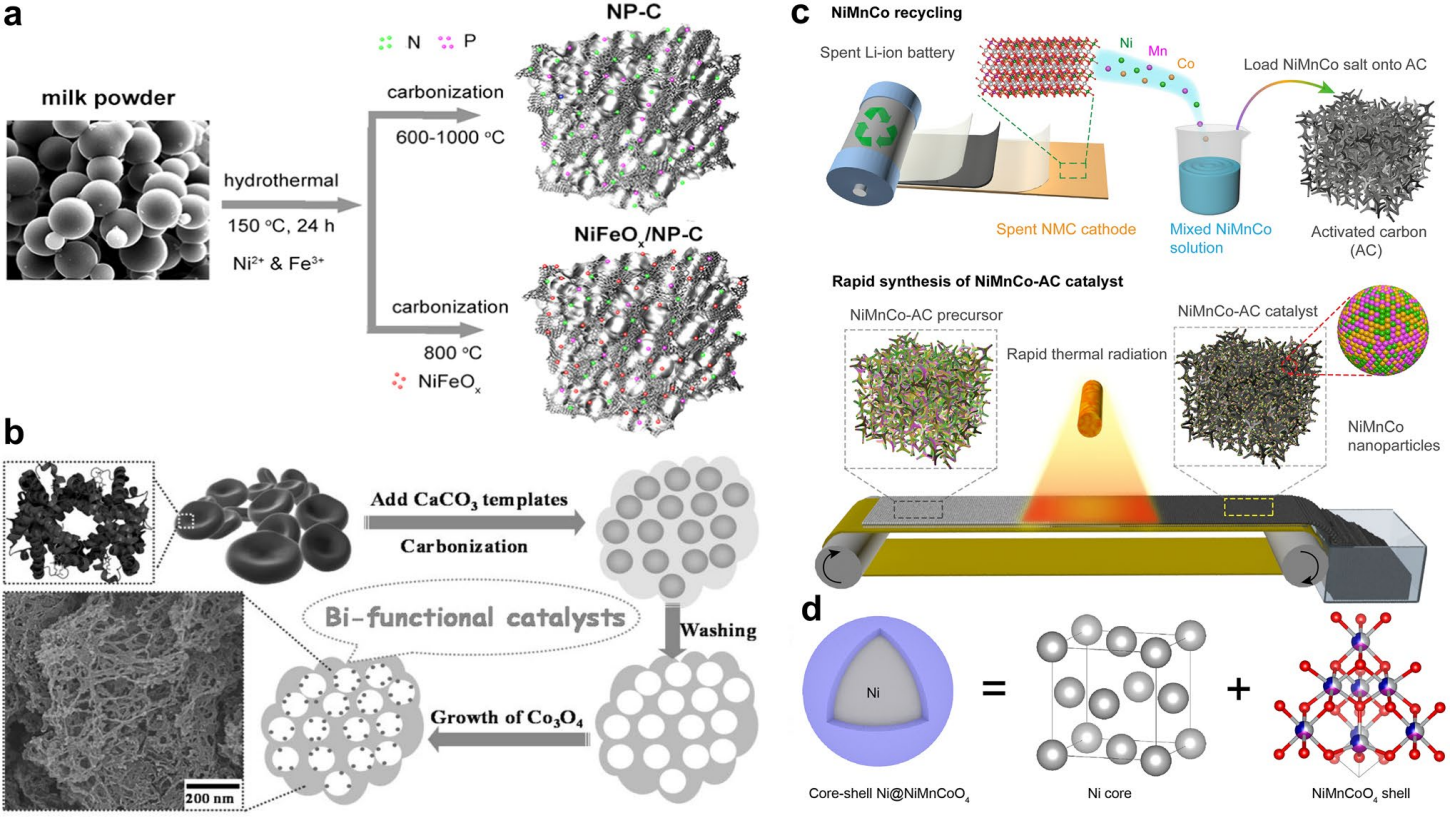
**a** Scheme of the preparation of milk powder-derived NP-C and NiFeO_x_/NP-C catalysts [163]. Copyright 2018, Elsevier. **b** Illustration of the synthesis of Co_3_O_4_ decorated BDHC [164]. Copyright 2014, Wiley–VCH. **c** Scheme of the preparation of the NiMnCo-AC electrocatalyst from spent Li-ion batteries. **d** Core–shell model of the NiMnCo nanoparticle [165]. Copyright 2022, National Academy of Sciences, USA

Loading metal (hydr)oxides on carbon scaffolds attracts growing interest in OER catalyst design, and the main reason is that the conductive carbon can effectively compensate for the relatively low conductivity of metal (hydr)oxides [162]. The OER performance of metal (hydr)oxides/carbon heterostructures can be optimized by regulating the external and internal properties of both metal (hydr)oxides and carbon materials. With a hydrothermal treatment-carbonization process, Chen et al. incorporated NiFeO_x_ nanoparticles (~ 10 nm) into N, P co-decorated carbon derived from milk powder (Fig. 10a) [163]. Benefiting from porous carbon’s large surface area and NiFeO_x_ nanoparticles’ high activities, the NiFeO_x_/carbon hybrid delivers a good OER activity. To regulate the nanostructure of carbon substrate, Zhang and co-authors proposed a CaCO_3_-involved approach to synthesize Co_3_O_4_/heteroatom-doped carbon catalysts (Fig. 10b) [164]. It is interesting to find that using CaCO_3_ as the template and activator leads to a unique fibrous network structure. The carbon material with a large surface area and rich heteroatom dopants can provide abundant anchoring sites for Co_3_O_4_, which significantly limit particle growth and aggregation and also improve the charge transfer process. Moreover, the intimate contact of Co_3_O_4_ and the carbon support leads to synergistic effects for OER.

Regulating metal (hydr)oxides’ properties can directly alter the catalytic performance of metal (hydr)oxides/carbon heterostructures. For instance, the intercalated borates in the hierarchical NiFe-borate LDH/N-doped carbon catalyst play a positive effect on the OER performance by improving the hydrophilicity, enlarging the surface area, populating electroactive sites, and providing abundant mass/charge transport pathways [93]. Alternatively, engineering a metal/oxide heterostructure on carbon materials is suggested to enhance the catalytic performance by the strong electronic interactions between the electroactive metals and oxides. Jiao et al. proposed a rapid thermal radiation strategy for transforming spent Li-ion batteries into a NiMnCo/carbon catalyst (NiMnCo-AC) for OER (Fig. 10c) [165]. Detailed characterizations suggest that the NiMnCo nanoparticles show a Ni@NiMnCoO_4_ core–shell nanostructure, including spinel NiMnCoO_4_ shell and fcc-structured Ni core (Fig. 10d). Further DFT calculations suggest that the charge density redistribution at the Ni/ NiMnCoO_4_ interface induced by Ni core and rich electroactive sites on the NiMnCoO_4_ shell ensure good OER performance.

**Fig. 11:**
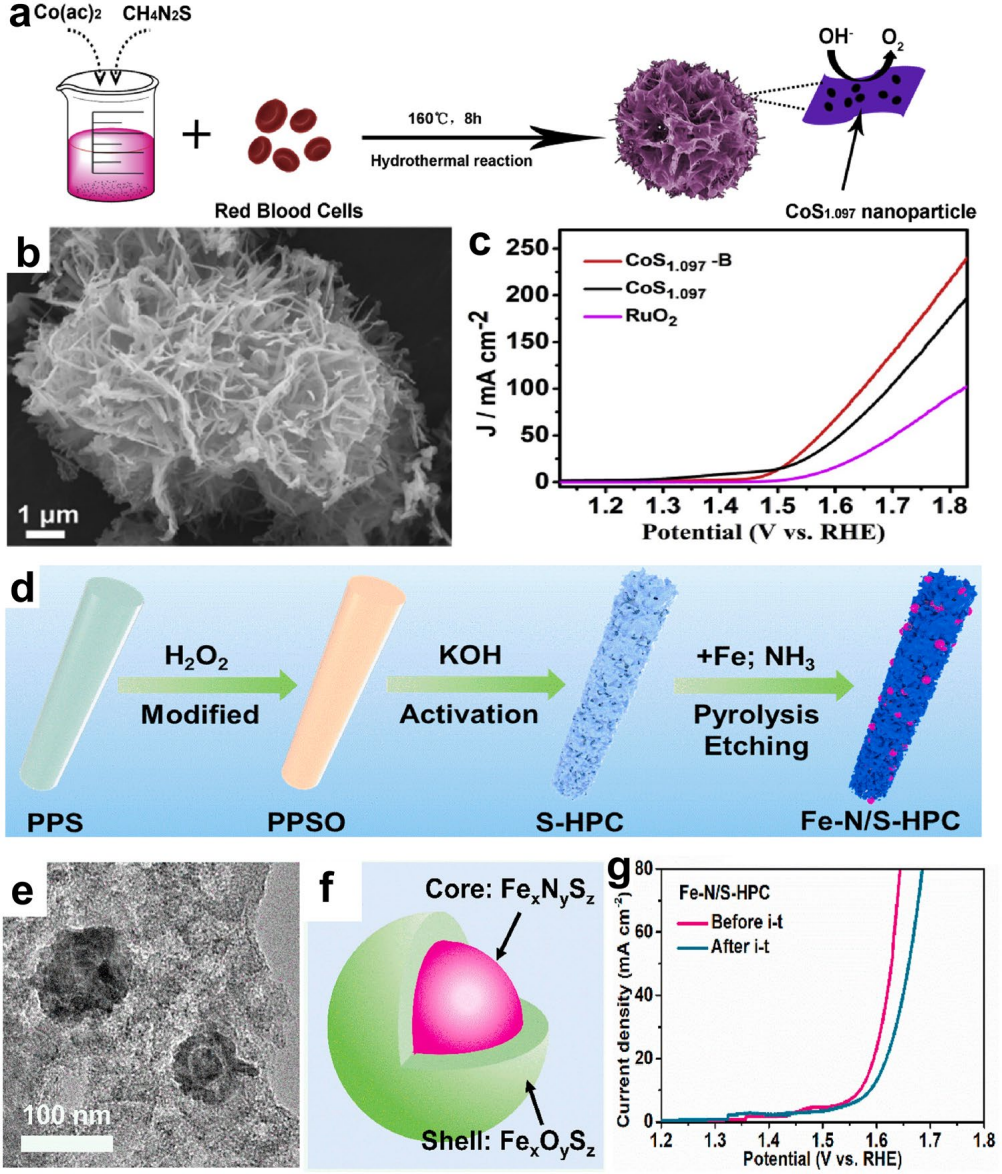
**a** Schematic of the fabrication of CoS_1.097_-B catalyst. **b** SEM image of CoS_1.097_-B catalyst. **c** LSV curves of catalysts in 1.0 M KOH [167]. Copyright 2020, Elsevier. **d** Scheme of Fe–N/S-HPC preparation from polyphenylene sulfide (PPS) plastic. **e** Transmission electron microscopy (TEM) image of Fe–N/S-HPC. **f** A model of Fe-based nanoparticles in Fe–N/S-HPC. **g** LSV curves of Fe–N/S-HPC before and after the 15 h OER durability test [169]. Copyright 2022, Elsevier

Carbon materials coupled with metal sulfides/nitrides/carbides/phosphides/borides heterostructures developed from wastes are promising OER catalysts. Compared with metal (hydr)oxides, the main merit of metal sulfides, nitrides, carbides, phosphides, and borides is their better electrical conductivity. Recent studies have emphasized the rational design of these metal compounds/carbon hybrids from wastes. Using dye sludge as the carbon source, Peng and coworkers developed a ZnS-involved N, S co-decorated carbon (ZnS/NSC) via a ZnCl_2_-involved pyrolysis process [166]. As suggested, the better OER performance of the ZnS/NSC catalyst prepared at a higher temperature (1000 °C) is due to its higher relative content of ZnS in the hybrid than catalysts synthesized at lower temperatures (800 and 900 °C). To improve the catalytic activity of CoS, a biowaste-derived carbon was introduced via a hydrothermal process (Fig. 11a) [167]. The obtained composite shows a flower-like (Fig. 11b) structure which enables high surface area, abundant active sites, and enhanced diffusion kinetics. It also can be seen that the nanoflower structure can provide abundant acute geometry at the nanoscale. Such features are favorable for concentrating the localized electric field at tips and providing enhanced adsorption of reaction intermediates, which would enhance the reaction kinetics [168]. Hence, the sulfide/carbon catalyst outperforms the bare sulfide and the RuO_2_ catalyst for OER (Fig. 11c).

Another method that enables upgrading sulfides/carbon catalysts’ OER performance is constructing electroactive hybrids on carbon materials. For example, Jiang et al. designed Fe_3_O_4_/NiS heterostructures on free-standing fibrous carbon (Fe_3_O_4_/NiS@C) [74]. The ternary composite shows excellent OER performance (*η*_10_ = 310 mV) and stability (no current density loss after 26 h OER operation) owing to the synergistic effect between electroactive Fe_3_O_4_ and NiS, as well as the self-standing hierarchically porous carbon structure. Using the sulfur-rich polyphenylene sulfide as the precursor, a Fe, N, S co-decorated porous carbon (Fe–N/S-HPC) was fabricated via a three-step route (Fig. 11d) [169]. The obtained Fe–N/S-HPC catalyst exhibits a honeycomb-like structure, and it is visible that rich spherical Fe particles are disseminated in the carbon matrix (Fig. 11e). Further characterizations suggest an electroactive Fe_x_N_y_S_z_@Fe_x_O_y_S_z_ structure (Fig. 11f), which can significantly enhance the OER activity and durability. As displayed in Fig. 11g, Fe–N/S-HPC reserves a high OER activity after the electrochemical stability test for 15 h.

## Waste-Derived Bifunctional Catalysts for OWE

Developing bifunctional electrocatalysts for OWE is of great significance in light of system simplification, cost reduction, and large-scale application of electrolyzers [170–172]. Encouragingly, many waste-derived catalysts deliver high activities toward both HER and OER (Table 3) and such cost-effective bifunctional electrocatalysts profoundly push the development of green hydrogen production. In this part, recent waste-derived bifunctional electrocatalysts for OWE are discussed.

**Table 3:** Summary of representative waste-derived electrocatalysts for OWE

Waste	Catalyst	Electrolyte	E_10_^a^ (V)	Durability	Refs
Camellia flower	S-doped carbon	1.0 M KOH	1.76	24 h @ ~ 20 mA cm^−2^	[174]
Corn stalks	Few-layer N-doped porous carbon	1.0 M KOH	1.60	–	[173]
Rose flower	Ni-doped graphitic carbon	1.0 M KOH	1.64	24 h @ 1.64 V	[176]
Textile sludge	Fe, N co-doped carbon	1.0 M KOH	1.70	14 h @ 1.7 V	[177]
Battery industrial wastewater	NiCoMn LTHs	Alkaline wastewater^b^	1.58	24 h @ 1.6 V	[39]
Spent Li-ion batteries	Ni/Ni-Mn-Co–O	1.0 M KOH	1.62	25 h @ 1.58 – 1.66 V	[178]
Scrap copper wires	NiCoP/Cu	1.0 M KOH	1.59	24 h @ 10 mA cm^−2^	[77]
Cornstalks	β-Mo_2_C/C	1.0 M KOH	1.65	30 h @ 10 mA cm^−2^	[215]
Cotton fibers	Co/carbon tubes	1.0 M KOH	1.40	110 h @ 1.4 V	[179]
Miscanthus stems	Co/C	1.0 M KOH	1.45	120 h @ 50 mA cm^−2^	[47]
Duckweed	NiFe-alloy/N, S-doped carbon	1.0 M KOH	1.61	200 h @ 2 V	[181]
Alfalfa	NiFe/N, P, S co-doped carbon	1.0 M KOH	1.60	50 h @ 10 mA cm^−2^	[182]
Grapefruit peels	NiFe-alloy/ N-doped carbon	1.0 M KOH	1.63	–	[180]
Magnolia leaves	CoP/C	1.0 M KOH	1.56	24 h @ 1.59 V	[216]
Polysaccharide chitin	Co_2_P/N, P co-doped carbon	1.0 M KOH	1.65	10 h @ ~ 20 mA cm^−2^	[190]
Ginkgo leaves	Co_2_P@CoP/C	1.0 M KOH	1.63	1000 min @ 10 mA cm^−2^	[189]
Cauliflower leaves	Ni/NiO/N-doped carbon	0.1 M KOH	1.688	20 h @ 10—30 mA cm^−2^	[188]
Amaranth	Fe, N co-doped carbon	1.0 M KOH	1.53	30 h @ 1.53 V	[183]
Lotus leaves	Co/MoO_2_@N doped carbon	1.0 M KOH	1.629	48 h @ 10 mA cm^−2^	[186]
Holly leaves	Co-CoO/C	1.0 M KOH	1.77	–	[187]
Chicken feathers	Ni-Co oxides/C	1.0 M KOH	1.53	200 h @ 1.7 V	[185]
Willow catkins	Co_3_O_4_/N-doped hollow carbon	1.0 M KOH	1.74	20 h @ 1.74 V	[184]
Waste yeast	Cu_8_S_5_ decorated N, S co-doped porous carbon	1.0 M KOH	1.64	14 h @ 10 mA cm^−2^	[191]
Tissue paper	Co_9_S_8_@Co–N/C nanorods	1.0 M KOH	1.61	70 h @ 10 mA cm^−2^	[192]
Catkin	MoS_2_@NiOOH@C	1.0 M KOH	1.62	40 h @ 1.62 V	[194]
Willow catkins	NiFe LDH/(NiFe)S_x_/hollow carbon	1.0 M KOH	1.53	100 h @ 10 mA cm^−2^	[48]
Spent Li-ion batteries	CoN/graphene	1.0 M KOH	1.61	40 h @ 1.68 V	[195]

^a^E_10_: Applied voltage at the current density of 10 mA cm^−2^

^b^Alkaline wastewater: Wastewater with 1 M KOH

### Waste-Derived Carbon Catalysts for OWE

**Fig. 12:**
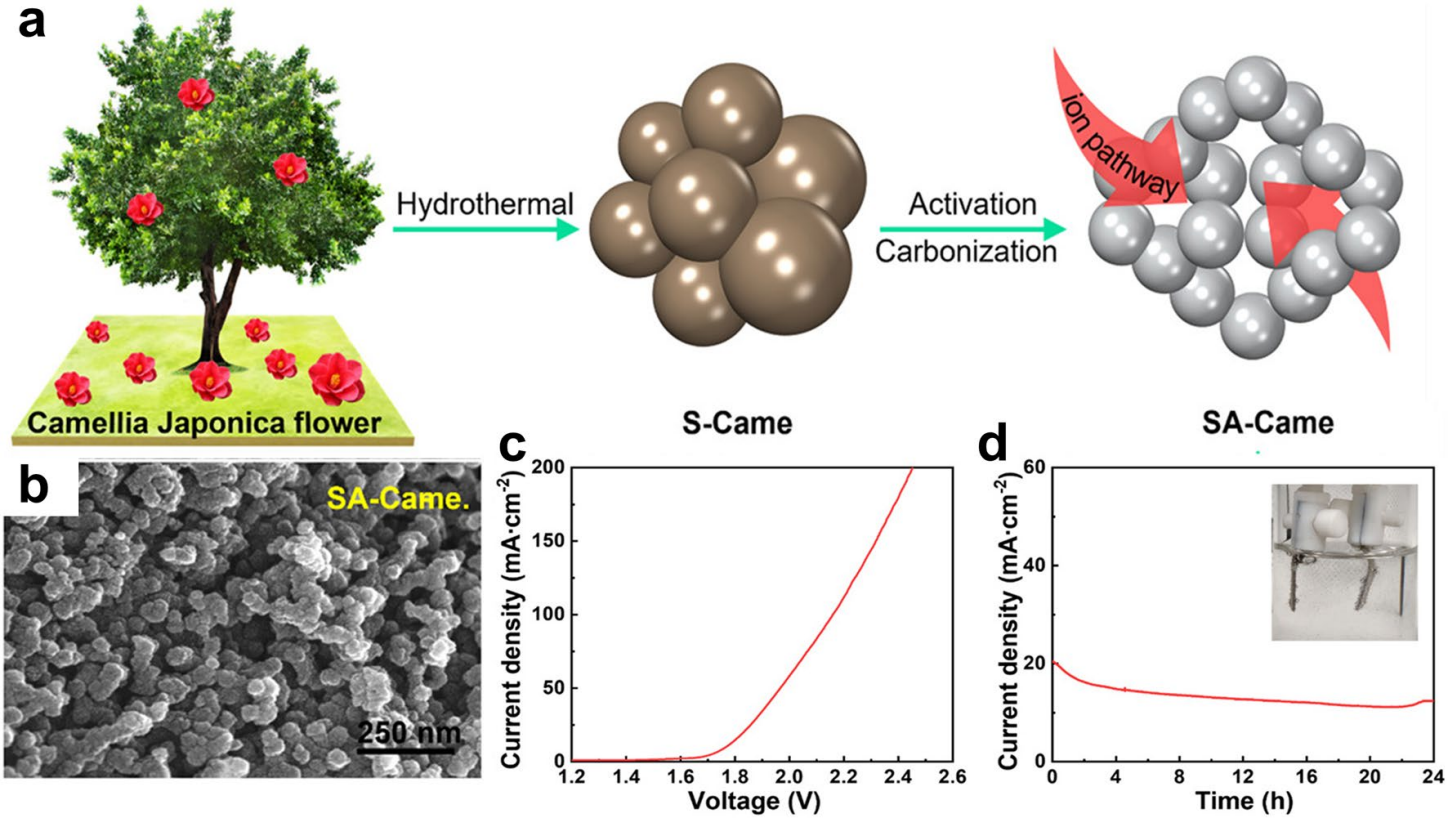
**a** Scheme of the preparation of SA-Came nanospheres. **b** SEM image of SA-Came. **c** LSV curve and **d** Chronoamperometry analysis of the SA-Came assisted water electrolyzer [174]. Copyright 2022, Wiley–VCH

Engineering the nanostructure and electronic properties of some heteroatom-doped carbon materials can catalyze HER and OER synchronically. Using the mixture of corn stalks soot and melamine as the precursor, Liu and co-authors developed a N-doped porous carbon electrocatalyst (NPCSS) for OWE [173]. With large SSA, abundant electroactive sites, and rich electrochemically active pyridinic/pyrrolic N species, NPCSS acquires 10 mA cm^−2^ at 1.60 V in a two-electrode cell. Besides N-doped carbon, S self-doped carbon also can catalyze water splitting. Xia et al. prepared the S self-doped activated camellia (SA-Came) carbon nanospheres from camellia flowers through a hydrothermal treatment–pyrolysis route (Fig. 12a) [174]. The obtained SA-Came catalyst shows a densely interconnected spherical morphology with a small particle size of approximately 50 nm (Fig. 12b). The rough surface and rich nanopores of the catalyst contribute to increased micropores and mesopores, which further enlarge the pore volume and surface area and lead to efficient mass/charge transfer during electrochemical processes. In addition, the abundant S sites induced more polarized surface domains with highly active sites, which benefit the electrocatalytic performance. To this end, the SA-Came catalyst requires a small *η* (0.53 V) to attain 10 mA cm^−2^ (Fig. 12c) with good performance stability for 24 h (Fig. 12d).

Incorporating active transitional metals into carbon materials is expected to attain enhanced catalytic performance [175]. For example, loading Ni onto the high graphitic carbon takes the advantage of graphitic carbon’s excellent electrical conductivity and the high electrocatalytic activity of Ni species [176]. In another study, Zhang et al. found that Fe species in the textile sludge facilitated the graphitization process of pyrolyzed 3D interconnected hierarchical Fe, N co-decorated carbon (TS–Fe–N–C) [177]. Combined with the high pyridinic-N content, uniformly distributed Fe-N_x_ and Fe_3_C electroactive sites, and hierarchical structure, the TS–Fe–N–C gains a high activity toward OWE (*E*_10_ = 1.70 V).

### Waste-Derived Transitional Metal-based Catalysts for OWE

**Fig. 13:**
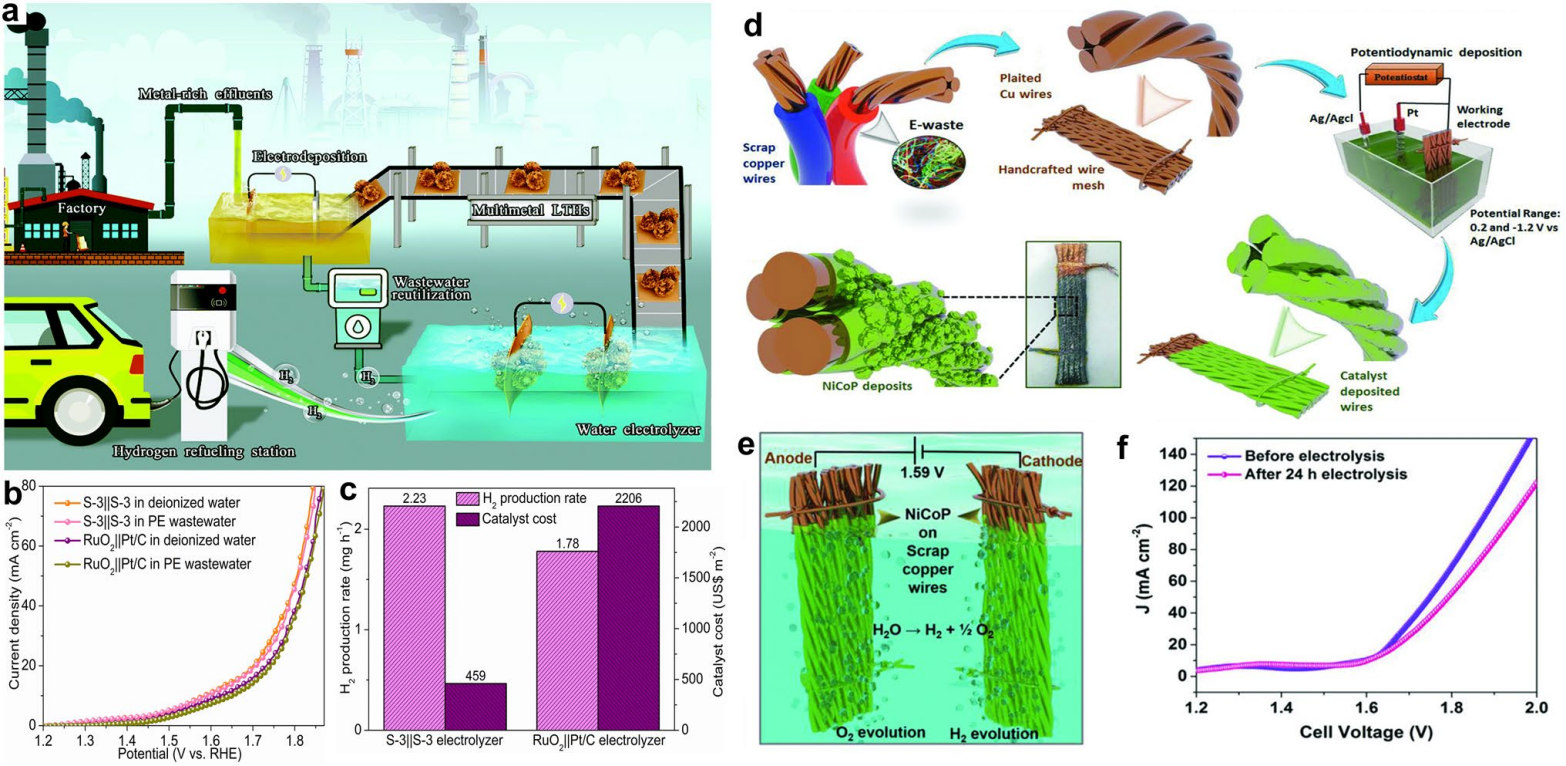
**a** Scheme of wastewater electrolyzed by wastewater-derived NiCoMn LTHs. **b** LSV curves of OWE of S-3||S-3 and RuO_2_||Pt/C in alkaline-deionized water and post-electrodeposition (PE) wastewater. **c** Comparison of hydrogen production rate and catalyst cost for the S-3||S-3 and RuO_2_||Pt/C PE wastewater electrolyzer systems [39]. Copyright 2022, Royal Society of Chemistry. **d** Schematic of the NiCoP/Cu electrode preparation. **e** Scheme of the OWE configuration. **f** LSV curves of NiCoP/Cu electrode before and after 24-h electrolysis [77]. Copyright 2018, Wiley–VCH

Transitional metals-rich wastes are highly desirable precursors for preparing bifunctional OWE electrocatalysts because of the high activity of transitional metals and low cost. For instance, Zheng et al. employed an ultrafast carbothermal shock method to transform the spent cathode of Li-ion batteries into a Ni/Ni-Mn-Co–O hybrid catalyst for OWE (*E*_10_ = 1.62 V) [178]. The co-presence of Ni-Mn-Co oxides and Ni metal ensures great conductivity and catalytic activity. Additionally, the hybrid’s small size and large electrochemically active surface area facilitate the exposure of abundant catalytic sites, promoting the mass/charge transfer process. Recently, our group has focused on the close-loop utilization of battery industrial wastewater with an electrodeposition-electrolysis route (Fig. 13a) [39]. In this process, the main metal ions (i.e., Ni, Co, Mn) have been converted into NiCoMn LTHs via electrodeposition, which shows favorable catalytic performance for OER and HER. The optimal deposit (S-3) possesses a hierarchical nanoflower structure that can act as a highly competitive electrocatalyst for post-electrodeposition (PE) wastewater electrolysis (Fig. 13b). The S-3||S-3-driven wastewater electrolyzer attains a higher hydrogen production efficiency at a much lower cost than the RuO_2_||Pt/C couple (Fig. 13c).

Besides the battery-based wastes-derived metal (hydr)oxides, the abundant waste Cu wires were used as a support to design phosphide-based bifunctional electrocatalysts for OWE [77]. Via electrodepositing highly active amorphous NiCoP films on the Cu wire, the obtained NiCoP/Cu hybrid shows high HER and OER activities. Using as a bifunctional catalyst, the NiCoP/Cu electrode attains 10 mA cm^−2^ at 1.59 V (Fig. 13e) and displays good stability in 24 h (Fig. 13f). These successful practices hint that it is convenient to engineer free-standing high-performance bifunctional electrocatalysts from metal-rich solid wastes/effluents.

### Waste-Derived Carbon-Based Heterostructures for OWE

To ameliorate transitional metal-based materials’ catalytic properties, introducing a highly conductive carbon support is usually implemented. In consequence, exploring eco-friendly and low-cost waste-derived carbon to prepare carbon/transitional metal-based materials heterostructures for OWE is greatly attractive for advancing efficient water electrolysis systems. Encouragingly, diverse transitional metal-based materials (e.g., metals, alloys, carbides, nitrides, oxides, sulfides, and phosphides) have been successfully coupled with biowaste-derived carbon, and the formed hybrids exhibit high performance toward OWE.

Loading highly electroactive and conductive metals or alloys within the carbon matrix is suggested to attain all-around performance for OWE [47]. Recently, Jiang and co-authors developed Co particles/biomass carbon tubes (Co-BCTs) catalysts from cotton fibers [179]. Using as the bifunctional electrocatalyst for OWE, the CO-BCTs can deliver 10 mA cm^−2^ at an applied potential of 1.40 V. Detailed characterizations imply the tight connection of Co particles with BCTs enhances conductivity and electron transfer kinetics, while BCTs’ loosely hierarchical structure facilitates mass/charge transport and sustains high stability. Apart from metal nanoparticles, Son et al. introduced Co single atoms, nanoclusters, and nanoparticles to a waste-derived self-standing carbon material with interconnected fibrous networks [47]. The multi-sized Co species decorated carbon catalyst shows high performance for OWE (*E*_10_ = 1.45 V), and the origin of electrochemical activities for different reactions has been disclosed. Specifically, the co-presence of Co single atoms and nanoparticles mainly contributed to the OER activity, while the principal electroactive sites for HER should be Co–N_x_ sites. NiFe-alloys hybridized N-doped graphene-like carbon [180], N, S co-doped mesoporous carbon [181], and N, P, S tri-doped nanocarbon [182] are also active toward OWE.

Metal oxides/carbon heterostructures can benefit from carbon’s high surface area and excellent conductivity and metal oxides’ high catalytic activity, thus forming efficient OWE catalysts. For instance, FeO_x_ [183] and Co_3_O_4_ [184] decorated N-doped hierarchical porous carbon have been reported as bifunctional electrocatalysts for OWE. To upgrade monometallic oxides’ intrinsic activity, recent studies have designed bimetallic oxides and metal/metal oxide hybrids on carbon supports. Compared with the NiCoO_x_/carbon hybrid, the electrochemical activities of CoO_x_/carbon and NiO_x_/carbon are lower for OWE [185]. This is because the co-presence of Ni and Co can provide more catalytically active sites than its monometallic analogues. Recently results implied that synergistic effects between metal/metal oxides could boost metal oxides/carbon hybrids’ electrochemical properties [186–188]. In an efficient bifunctional Co–CoO nanoparticles/porous carbon catalyst, multiple active sites are involved for OWE, including Co–CoO, Co–N–C, and N-doped carbon [187]. Of note, a high N content (primarily graphitic-N, pyridinic-N) in the carbon can regulate the charge distribution of adjacent carbon atom and improve the hydrophobicity of catalysts. Additionally, the rich Co–CoO nanoparticles with strong synergistic effects can provide highly active sites toward both HER and OER, thereby realizing high catalytic activities.

**Fig. 14:**
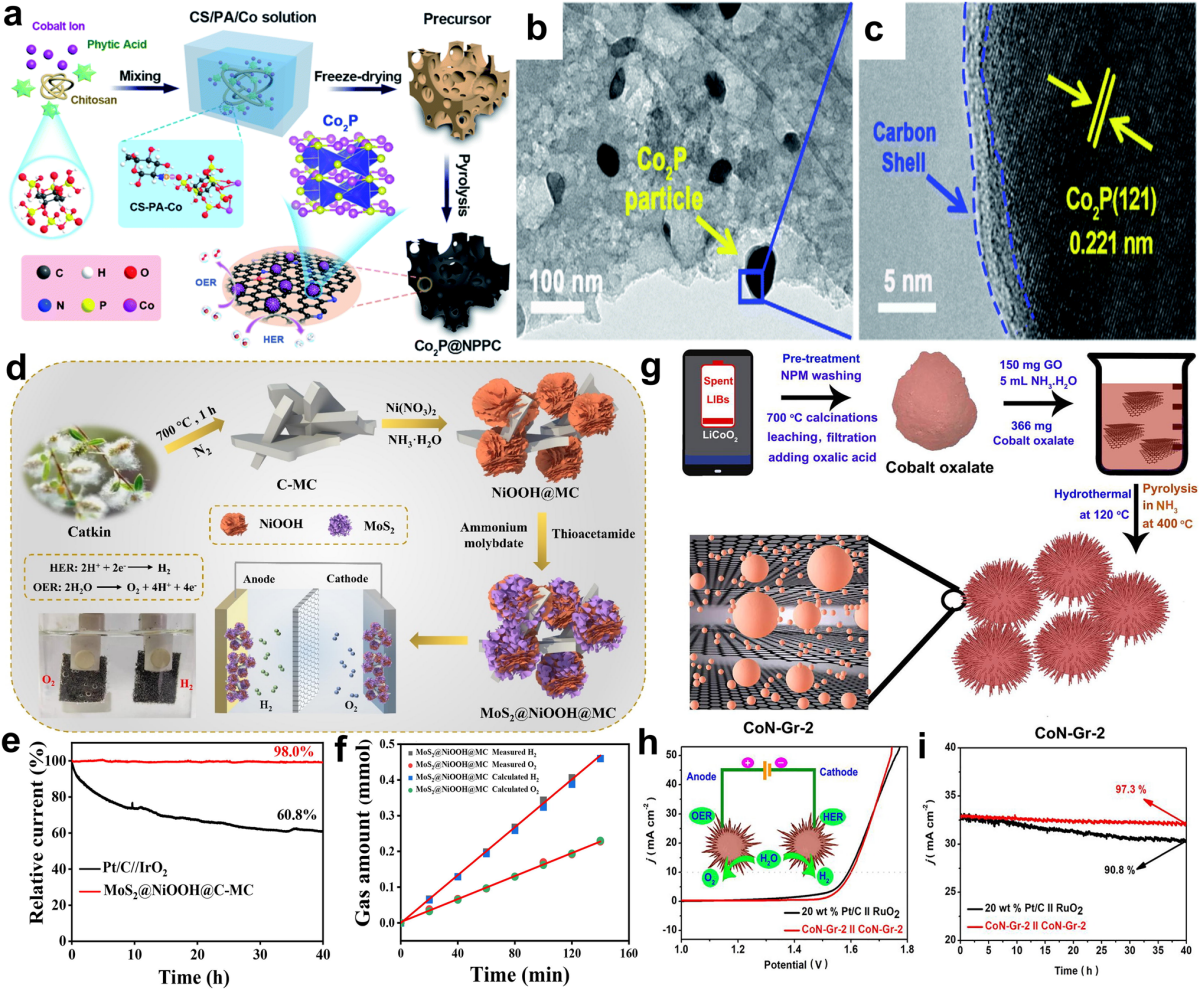
**a** Diagram of the preparation of Co_2_P@NPPC. **b** TEM and **c** high resolution TEM (HRTEM) images of Co_2_P@NPPC [190]. Copyright 2021, Royal Society of Chemistry. **d** Scheme of the synthesis of the MoS_2_@NiOOH@C-MC composite. **e** Stability test of the Pt/C||IrO_2_ and MoS_2_@NiOOH@C-MC||MoS_2_@NiOOH@C-MC couples for OWE, at 10 mA cm^−2^. **f** The time-dependent of the experimental and theoretical H_2_ and O_2_ production amounts during OWE using the MoS_2_@NiOOH@C-MC catalyst [194]. Copyright 2022, Elsevier. **g** Scheme of the preparation of tiny CoN-coupled graphene hybrid (CoN-Gr-2). **h** The OWE performance of the CoN-Gr-2||CoN-Gr-2 and Pt/C||RuO_2_ couples. **i** Stability test of CoN-Gr-2 for OWE in 1.0 M KOH at 1.68 V [195]. Copyright 2021, Elsevier

Metal phosphides are promising bifunctional electrocatalysts for OWE, and a feasible way to further upgrade metal phosphides’ catalytic performance is by coupling them with porous carbon materials [189]. Starting from natural polysaccharide chitin, Li et al. designed a metal phosphide-based core–shell hybrid for OWE, which is composed of Co_2_P core and N, P co-decorated porous carbon shell (Co_2_P@NPPC) (Fig. 14a) [190]. Abundant Co_2_P nanoparticles are well isolated and fixed in the porous carbon, which can enhance electron transfer, expose rich active sites, and keep good stability for catalytic reactions (Fig. 14b-c). In another bifunctional Co_2_P@CoP/N, S co-doped carbon hybrid, the role of each component in catalyzing OWE has been uncovered by Lin and coworkers [189]. Specifically, the synergistic effects of the Co_2_P@CoP heterostructure make a near-zero ΔG_H*_, resulting in a high HER activity. Also, the Co_2_P@CoP hybrid causes the conduction band to bend downwards, leading to high OER activity. Moreover, N and S dopants can tune the carbon support’s electronic property, and sustain the suitable electron-donating feature to enhance overall electrocatalytic properties.

Metal sulfides/carbon heterostructures have been widely designed from wastes for OWE. For instance, the textile sludge derived Cu_8_S_5_/N, S co-doped porous carbon [191], and the Co_9_S_8_/carbon nanorod framework (Co_9_S_8_@Co–N/C) fabricated from waste tissue paper towel [192] are highly efficient bifunctional catalysts. Constructing electroactive hybrids on carbon support is a universal method to boost the performance of metal sulfides/carbon heterostructures [48]. Typically, MoS_2_ is an active HER catalyst with low OER performance [193]. To make a bifunctional catalyst with MoS_2_, it is necessary to incorporate an OER active component. Liu et al. developed the MoS_2_@NiOOH hybrid on mesoporous carbon support synthesized from catkin (MoS_2_@NiOOH@C-MC) via a three-step process (Fig. 14d) [194]. By combining the high OER activity of NiOOH, HER activity of MoS_2_, as well as C-MC’s efficient charge transfer kinetics, the multicomponent MoS_2_@NiOOH@C-MC performs better than the Pt/C||IrO_2_ couple for OWE regarding catalytic activity and performance durability (Fig. 14e). Also, the high reaction Faraday efficiencies of 99.6% (HER) and 98.7% (OER) further evidence the excellent catalytic performances of the catalyst toward OWE (Fig. 14f). Different from most carbon-based heterostructures made from biowastes, Liu and co-authors prepared a CoN/graphene composite from spent Li-ion batteries (Fig. 14g) [195]. Benefiting from the high intrinsic conductivity and activity, sea–urchin-like nanostructure, and high surface area, the optimal sample (CoN-Gr-2) shows comparable performance to the Pt/C||IrO_2_ couple for OWE, with better stability for 40 h at 1.68 V (Fig. 14h, i).

## Conclusions and Perspectives

Following circular economy principles, engineering-efficient electrocatalysts from wastes for water electrolysis is of great environmental and economic benefits. In this review, recent achievements in the design of waste-based catalysts for HER, OER, and OWE have been systematically analyzed. Diverse wastes (especially biowastes and electronic wastes) have been successfully converted into electrocatalysts via pyrolysis, electrochemical synthesis, wet-chemical synthesis, microwave synthesis, etc. The waste-derived carbon-based catalysts, transitional metal-based catalysts, and carbon-based heterostructures have exhibited good performance toward HER, OER, and OWE. Catalysts’ performance is highly related to their nanostructure, chemical composition, and electronic property, which can be regulated by waste precursors and synthesis methods.

Despite these exciting scientific achievements, many opportunities implore further investigations in this expanding field.

1. Exploring diverse wastes for the design of high-performance electrocatalysts. Currently, most studies focus on biowastes and some electronic wastes (mainly spent batteries), while other types of wastes (e.g., plastic wastes, liquid wastes) are still less explored. More attention should be paid to the reutilization of ever-growing plastic wastes due to their high carbon content, large quantity, and hazardous effect on the ecosystem. Since heteroatom-doped carbon is more active than pure carbon materials for electrochemical applications, it is better to choose biowastes/plastic wastes with a high content of non-carbon elements (e.g., N, P, S, B) as the catalyst precursors. In addition, it is a sensible way to co-treat biowastes/plastic wastes and electronic wastes to form transitional metal materials/carbon heterostructures which have shown favorable catalytic performance toward HER, OER, and OWE.
2. It is crucial to adopt advanced techniques to gain clear and fundamental insights into the origin of electrochemical activity. Most wastes present complicated compositions and structures, which causes many challenges in catalytic mechanism investigations and the reproducibility of research. To this end, integrating advanced analytical, electrochemical, microscopic, spectroscopic, and computational techniques to investigate the composition-structure-catalytic performance relationship would guide the design of high-efficiency electrocatalysts. Importantly, easily overlooked defects, dopants, and single-atom sites in waste-derived catalysts should be checked carefully, because these features can profoundly govern the catalytic performance.
3. To realize large-scale production of catalysts from wastes, facile and low-cost fabrication techniques are required. Considering the environmental impacts, catalyst preparation processes with limited carbon emissions and low energy consumption are highly suggested. Some techniques like electrodeposition, ball milling, plasma synthesis, and flash Joule heating are favorable options. Importantly, it is suggested to perform a pre-treatment process to remove hazardous and toxic substances (e.g., radioactive elements) in some typical wastes before the preparation and utilization of waste-derived catalysts. In a circular economy view, it is feasible to recover and reuse the spent waste-derived electrocatalysts for further applications.
4. Waste-derived electrocatalysts have shown promising performance for water electrolysis, but they are still far from satisfactory. To further improve the catalytic performance of waste-derived catalysts, advanced strategies are encouraged to improve the intrinsic catalytic activity, electroactive sites, mass/charge transfer, mechanical and electrochemical stability of catalysts. In this context, implementing sophisticated methods (e.g., heteroatom doping, nanostructure design, defect engineering, heterostructure construction, and crystallinity control) to synergistically regulate catalysts’ internal and external characteristics would meet the necessities for waste-derived electrocatalysts toward practical water electrolysis.
5. Considering the high redox property and low cost of waste-derived catalysts, it is of great environmental and economic value to implement waste-derived catalysts in other electrochemical reactions related to environmental remediation and energy storage/conversion, such as nitrogen/nitrate reduction, organic pollutant oxidation/reduction, oxygen reduction, carbon dioxide reduction, hydrogen oxidation, and biomass oxidation. The wide application of waste-derived catalysts would help to minimize the carbon footprint of functional materials preparation and largely facilitate waste management.
